# ClusterMatch aligns single-cell RNA-sequencing data at the multi-scale cluster level via stable matching

**DOI:** 10.1093/bioinformatics/btae480

**Published:** 2024-07-29

**Authors:** Teer Ba, Hao Miao, Lirong Zhang, Caixia Gao, Yong Wang

**Affiliations:** School of Physical Science and Technology, Inner Mongolia University, Hohhot 010021, China; School of Mathematical Sciences, Inner Mongolia University, Hohhot 010021, China; CEMS, NCMIS, HCMS, MDIS, Academy of Mathematics and Systems Science, Chinese Academy of Sciences, Beijing 100190, China; School of Mathematics, University of Chinese Academy of Sciences, Chinese Academy of Sciences, Beijing 100049, China; School of Physical Science and Technology, Inner Mongolia University, Hohhot 010021, China; School of Mathematical Sciences, Inner Mongolia University, Hohhot 010021, China; CEMS, NCMIS, HCMS, MDIS, Academy of Mathematics and Systems Science, Chinese Academy of Sciences, Beijing 100190, China; School of Mathematics, University of Chinese Academy of Sciences, Chinese Academy of Sciences, Beijing 100049, China; Center for Excellence in Animal Evolution and Genetics, Chinese Academy of Sciences, Kunming 650223, China; Key Laboratory of Systems Biology, Hangzhou Institute for Advanced Study, University of Chinese Academy of Sciences, Chinese Academy of Sciences, Hangzhou 330106, China

## Abstract

**Motivation:**

Unsupervised clustering of single-cell RNA sequencing (scRNA-seq) data holds the promise of characterizing known and novel cell type in various biological and clinical contexts. However, intrinsic multi-scale clustering resolutions poses challenges to deal with multiple sources of variability in the high-dimensional and noisy data.

**Results:**

We present ClusterMatch, a stable match optimization model to align scRNA-seq data at the cluster level. In one hand, ClusterMatch leverages the mutual correspondence by canonical correlation analysis and multi-scale Louvain clustering algorithms to identify cluster with optimized resolutions. In the other hand, it utilizes stable matching framework to align scRNA-seq data in the latent space while maintaining interpretability with overlapped marker gene set. Through extensive experiments, we demonstrate the efficacy of ClusterMatch in data integration, cell type annotation, and cross-species/timepoint alignment scenarios. Our results show ClusterMatch’s ability to utilize both global and local information of scRNA-seq data, sets the appropriate resolution of multi-scale clustering, and offers interpretability by utilizing marker genes.

**Availability and implementation:**

The code of ClusterMatch software is freely available at https://github.com/AMSSwanglab/ClusterMatch.

## 1 Introduction

The cell is the basic structural and functional unit of all forms of life (Harris 2000). In recent years, advancements in single-cell sequencing technologies have enabled researchers to access cellular information at the individual cell level (Kelsey *et al.* 2017, Nagano *et al.* 2013, Rotem *et al.* 2015, Satpathy *et al.* 2019, Smallwood *et al.* 2014), which brings rich characterization for known and novel cell types with price of increasing statistical uncertainty. Among these techniques, single-cell RNA sequencing (scRNA-seq) has emerged as a powerful tool for profiling numerous single-cell transcriptomes across various tissues, organs, and species (Klein *et al.* 2015, Picelli *et al.* 2013, Ramsköld *et al.* 2012, Svensson *et al.* 2018, Zheng *et al.* 2017). A substantial volume of scRNA-seq data has been amassed, encompassing diverse species, tissues, and health conditions (Arazi *et al.* 2018, Cao *et al.* 2020, Shami *et al.* 2020). To reduce the uncertainty in the heuristic un-supervised clustering analysis, it has become crucial to align and integrate different scRNA-seq datasets to borrow information and analyze the differences and similarities of the datasets. For instance, comparing scRNA-seq data from distinct species allows for the identification of conserved and specific cell types, shedding light on evolutionary mechanisms (Shafer 2019). Similarly, contrasting scRNA-seq data from different disease states unveils gene expression patterns and provides insights into the diseases pathogenesis (Laughney *et al.* 2020).

From methodology perspective, the alignment of scRNA-seq data is a crucial step for the downstream analysis (Haghverdi *et al.* 2018, Luecken and Theis 2019, Luecken *et al.* 2022, Ryu *et al.* 2023, Stuart *et al.* 2019) such as cluster annotation, label transfer, and trajectory analysis (Cao *et al.* 2019, Luecken and Theis 2019, Stuart *et al.* 2019). However, aligning scRNA-seq data poses challenges due to its high-dimension and high-noise characteristics (Luecken and Theis 2019), as well as the presence of batch effects between different datasets (Haghverdi *et al.* 2018, Luecken *et al.* 2022). To overcome these obstacles, existing approaches first commonly use dimensionality reduction methods as the initial step to project cells from different batches into a common low-dimensional space. Various methods have been developed to achieve dimensionality reduction for scRNA-seq data alignment. For instance, fastMNN (Haghverdi *et al.* 2018), BBKNN (Polański *et al.* 2020), Conos (Barkas *et al.* 2019), and Harmony (Korsunsky *et al.* 2019) utilize principal component analysis (PCA), Seurat V3 (Stuart *et al.* 2019) utilizes canonical correlation analysis (CCA), LIGER (Welch *et al.* 2019) uses integrative nonnegative matrix factorization (iNMF), and DESC (Li *et al.* 2020), scVI (Lopez *et al.* 2018) and scANVI (Xu *et al.* 2021) use autoencoders. These dimensionality reduction techniques transform the original high-dimensional and noisy expression matrix into a low-dimensional space enriched with relevant signals. Following dimensionality reduction, the alignment of scRNA-seq data is accomplished by identifying similar cells at either the individual cell level or the cluster level using the projection values obtained from the aforementioned methods. Secondly, most alignment methods rely on nearest neighbor-based approaches at individual cell level, including MNN (Haghverdi *et al.* 2018), Seurat V3 (Stuart *et al.* 2019), BBKNN (Polański *et al.* 2020), and Conos (Barkas *et al.* 2019). Some methods align scRNA-seq data at the cluster level, such as Harmony (Korsunsky *et al.* 2019), DESC (Li *et al.* 2020), and LIGER (Welch *et al.* 2019). Harmony utilizes an iterative algorithm that first clusters similar cells using soft *k*-means clustering [*k* = min (100, M/30), where M represents the total number of cells]. It then aligns scRNA-seq data by utilizing the centroids of the clusters and the centroids specific to each cluster’s dataset. LIGER and DESC apply the Louvain clustering algorithm (Blondel *et al.* 2008) to cluster cells in the low-dimensional space. They subsequently use these clusters to align scRNA-seq data at the cluster level.

Although existing methods have made progress in aligning scRNA-seq data, there are still challenges to overcome. First, single cell clustering is intrinsic multi-scale and careful consideration must be given to the level of alignment: Which cell population is not happen by chance and what is the mutually optimal cluster level (Ryu *et al.* 2023). This decision carries implications for the accuracy and interpretability of the alignment process. Current methods do not consider this question. Second, when aligning scRNA-seq data at the cluster level, the representation of clusters, determination of their number, and ensuring their biological interpretability pose substantial challenges and captivate the interest of biologists (Korsunsky *et al.* 2019, Luecken and Theis 2019, Ryu *et al.* 2023, Stuart *et al.* 2019). Third, the global and local information of the data should be fully balanced for batch removal and biological variance conservation (Luecken *et al.* 2022, Tran *et al.* 2020, Wang *et al.* 2022) in dimensionality reduction. The widely used Seurat V3 (Stuart *et al.* 2019) uses CCA for dimensionality reduction, prioritizing global information to remove batch effects but potentially overlooking local information within each dataset, consequently disregarding important biological differences (Luecken *et al.* 2022, Tran *et al.* 2020, Wang *et al.* 2022).

**Figure 1.**
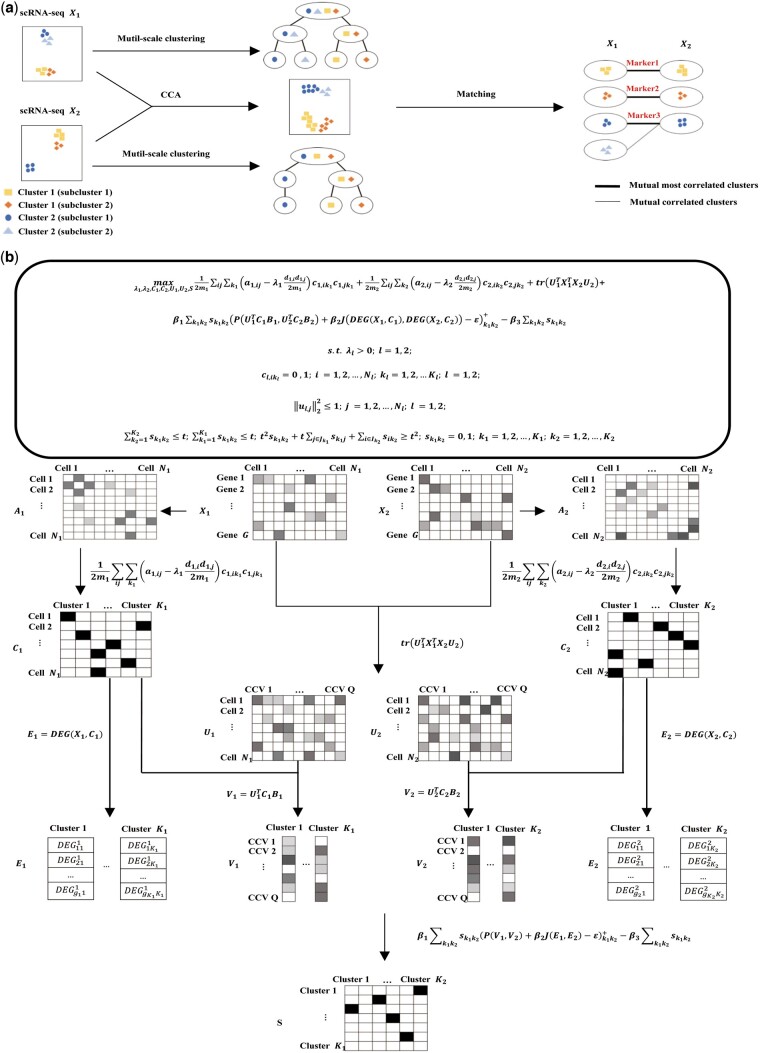
Overview of the ClusterMatch method for aligning two scRNA-seq datasets. (**a**) ClusterMatch uses stable matching to align the scRNA-seq data at the cluster level and clusters are identified by multi-scale Louvain clustering algorithms with optimized resolutions and represented by CCA and its associated marker gene set. (**b**) A graphical illustration for the algorithm to output the cluster alignment result S from the input data $X_{1}$ and $X_{2}$. The three major components are multi-scale clustering with optimized resolution, dimensional reduction by CCA, and stable matching. (i) $\frac{1}{2m_{l}}\sum_{ij}\sum_{k_{l}}\left(a_{l,ij}-\lambda_{l}\frac{d_{l,i}d_{l,j}}{2m_{l}}\right)c_{l,ik_{l}}c_{l,jk_{l}}$; l=1,2: $c_{l,ik_{l}}$ represents the assignment of the i-th cell to the $k_{l}$-th cluster in $X_{l}$ by maximizing the multi-scale modularity. $a_{l,ij}$ denotes the similarity between the i-th cell and the j-th cells in $X_{l}$, and expected edge weights are calculated as $\frac{d_{l,i}d_{l,j}}{2m_{l}}$, where $d_{l,i}=\sum_{j=1}^{N_{l}}a_{l,ij}$ is the sum of edge weights connected to the i-th cell and $m_{l}=\frac{1}{2}\sum_{i}^{N_{l}}\sum_{j}^{N_{l}}a_{l,ij}$ is the total weight of the edges in $X_{l}$. $\lambda_{l}$ is introduced as variable to optimize the clustering resolution; (ii) $tr\left(U_{1}^{T}X_{1}^{T}X_{2}U_{2}\right):U_{1}$ and $U_{2}$ provide low-dimensional representations of the cells in $X_{1}$ and $X_{2}$, respectively, obtained by CCA. (iii) $\beta_{1}\sum_{k_{1}k_{2}}s_{k_{1}k_{2}}{\left(P\left(U_{1}^{T}C_{1}B_{1},U_{2}^{T}C_{2}B_{2}\right)+\beta_{2}J\left(\mathrm{DEG}\left(X_{1},C_{1}\right),\mathrm{DEG}\left(X_{2},C_{2}\right)\right)-\varepsilon \right)}_{k_{1}k_{2}}^{+}-\beta_{3}\sum_{k_{1}k_{2}}s_{k_{1}k_{2}}$: Each cluster is represented by a low-dimensional vector $U_{l}^{T}C_{l}B_{l}$ and a differentially expressed genes set $\mathrm{DEG}\left(X_{l},C_{l}\right)$. The correlation is calculated using the Pearson correlation coefficient (P) and Jaccard coefficient (J). $\beta_{3}\sum_{k_{1}k_{2}}s_{k_{1}k_{2}}$ constrains the number of cluster correspondence, which helps to determine the optimal value of $\lambda_{l}$. Together with the constraints and sparse regularization, this stable matching component aims for the mutually beneficial alignment result

Here, we present ClusterMatch, an optimization model designed for aligning scRNA-seq data at the mutually optimal cluster level (Fig. 1). Different clusters may represent distinct cell states or cell types (Luecken and Theis 2019). Notably, according to Waddington’s concept of the epigenetic landscape, attractor states, which exhibit robustness against random fluctuations, often correspond to cell states (Bhattacharya *et al.* 2011, Bocci *et al.* 2022, Ferrell 2012, Li *et al.* 2016, Waddington 2014). Therefore, aligning scRNA-seq data at the cluster level offers enhanced stability and reliability compared to alignment at the individual cell level. ClusterMatch uses CCA and multi-scale Louvain clustering algorithms (Blondel *et al.* 2008, Levine *et al.* 2015, Traag *et al.* 2019) to determine optimal resolutions for cluster corroborating each other in scRNA-seq data, and represent cluster by low dimensional embeddings and marker gene set. Subsequently, the stable matching algorithm (Haghverdi *et al.* 2018, Shimada *et al.* 2020) is utilized to align scRNA-seq data at the cluster level. We demonstrate the effectiveness of our method in multiple scenarios, including scRNA-seq data integration, cell type annotation, and cross-species/timepoint alignment. In the data integration scenario, ClusterMatch successfully balances global and local information, removing batch effects while conserving biological variance. In the cell type annotation scenario, ClusterMatch achieves higher accuracy in labeling cell types by leveraging well-annotated human datasets to annotate nonlabelled macaque data (Bakken *et al.* 2021). Finally, in the cross-species/timepoint alignment scenario, we analyze scRNA-seq data from sheep and cattle rumen, reveal six core conserved cell types, and construct a differentiation map (Wu *et al.* 2022, Yuan *et al.* 2022). Overall, our study introduces ClusterMatch as an optimized model for the alignment and analysis of scRNA-seq data at the cluster level. It demonstrates excellent performance in data integration, cell type annotation, and cross-species/timepoint alignment, allowing for accurate and interpretable knowledge extraction from scRNA-seq data.

## 2 Materials and methods

### 2.1 Data preprocessing

Our input raw data were two scRNA-seq matrices. To preprocess the raw data, we utilized the Seurat V3 (Stuart *et al.* 2019). Specifically, we applied the “NormalizeData” and “ScaleData” functions to normalize and scale the data, respectively. In addition, we used the “FindVariableFeatures” function to identify the top 2000 most variable genes. Subsequently, we performed PCA using the “RunPCA” function and determined the shared nearest neighbor (SNN) of each cell in the two datasets using the “FindNeighbors” function. The preprocessed datasets utilized in the ClusterMatch model included the scaled data $X_{1}\in R^{G\times N_{1}}$ and $X_{2}\in R^{G\times N_{2}}$, as well as SNN graphs $A_{1}\in R^{N_{1}\times N_{1}}$ and $A_{2}\in R^{N_{2}\times N_{2}}$, where G represents the number of highly variable genes that intersect between $X_{1}$ and $X_{2}$.

### 2.2 ClusterMatch model

ClusterMatch is an optimization model for aligning scRNA-seq data at the cluster level. We first introduced some notations for our input data matrices: (i) scaled scRNA-seq matrices: $X_{1}\in R^{G\times N_{1}}$ and $X_{2}\in R^{G\times N_{2}}$, where $x_{l,gi}$ denoted the expression level of the g-th gene in the i-th cell of the data $X_{l}$, l=1,2. (ii) SNN graphs: $A_{1}\in R^{N_{1}\times N_{1}}$ and $A_{2}\in R^{N_{2}\times N_{2}}$, where $a_{l,ij}$ denoted the similarity of the i-th cell and the j-th cell in the data $X_{l}$, l=1,2. To infer the cluster resolutions and the cluster matching matrix, we formulated the following optimization model.


(1) $$\begin{array}{l} \underset{\lambda_{1},\lambda_{2},C_{1},C_{2},U_{1},U_{2},S}{\max}\frac{1}{2m_{1}}\sum_{ij}\sum_{k_{1}}\left(a_{1,ij}-\lambda_{1}\frac{d_{1,i}d_{1,j}}{2m_{1}}\right)c_{1,ik_{1}}c_{1,jk_{1}}\\ +\frac{1}{2m_{2}}\sum_{ij}\sum_{k_{2}}\left(a_{2,ij}-\lambda_{2}\frac{d_{2,i}d_{2,j}}{2m_{2}}\right)c_{2,ik_{2}}c_{2,jk_{2}}+tr\left(U_{1}^{T}X_{1}^{T}X_{2}U_{2}\right)\\ +\beta_{1}\sum_{k_{1}k_{2}}s_{k_{1}k_{2}}(P\left(U_{1}^{T}C_{1}B_{1},U_{2}^{T}C_{2}B_{2}\right)\\ {+\beta_{2}J\left(\mathrm{DEG}\left(X_{1},C_{1}\right),\mathrm{DEG}\left(X_{2},C_{2}\right)\right)-\varepsilon )}_{k_{1}k_{2}}^{+}\\ -\beta_{3}\sum_{k_{1}k_{2}}s_{k_{1}k_{2}}\\ s.t.\;\lambda_{l}>0;\;l=1,2\\ c_{l,ik_{l}}=0,1;\;i=1,2,\ldots ,N_{l};\;k_{l}=1,2,\ldots K_{l};\;l=1,2\\ {\left\|u_{l,j}\right\|}_{2}^{2}\leq 1;\;j=1,2,\ldots ,N_{l};\;l=1,2\\ \sum_{k_{2}=1}^{K_{2}}s_{k_{1}k_{2}}\leq t;\;\sum_{k_{1}=1}^{K_{1}}s_{k_{1}k_{2}}\leq t;\;t^{2}s_{k_{1}k_{2}}+t\sum_{j\in J_{k_{1}}}s_{k_{1}j}+\sum_{i\in I_{k_{2}}}s_{ik_{2}}\geq t^{2};\;\\ s_{k_{1}k_{2}}=0,\;1;\;k_{1}=1,2,\ldots ,K_{1};\;k_{2}=1,2,\ldots ,K_{2} \end{array}$$

To explain this formulation, we briefly discussed each term in the objective function.

1.
$\frac{1}{2m_{l}}\sum_{ij}\sum_{k_{l}}\left(a_{l,ij}-\lambda_{l}\frac{d_{l,i}d_{l,j}}{2m_{l}}\right)c_{l,ik_{l}}c_{l,jk_{l}}$ ; l=1,2: ClusterMatch clusters data $X_{1}$ and data $X_{2}$ by maximizing the multi-scale modularity, which maximizes the difference between the actual weight of an edge in the cluster and the expected weight of that edge. $a_{l,ij}$ is the actual weight of the edge between the i-th cell and the j-th cell in data $X_{l}$. The expected weights of edges can be expressed as $\frac{d_{l,i}d_{l,j}}{2m_{l}}$, where $d_{l,i}=\sum_{j=1}^{N_{l}}a_{l,ij}$ is the sum of the weights of the edges connected to the i-th cell in data $X_{l}$. $m_{l}=\frac{1}{2}\sum_{i}^{N_{l}}\sum_{j}^{N_{l}}a_{l,ij}$ is the total weight of the edges in data $X_{l}$. $\lambda_{l}>0$ is the cluster resolution. $c_{l,ik_{l}}$ indicates whether the i-th cell belongs to the $k_{l}$-th cluster in data $X_{l}$ or not. If it belongs to the $k_{l}$-th cluster, $c_{l,ik_{l}}=1$, otherwise $c_{l,ik_{l}}=0$. Notably, by fixing $\lambda_{l}$ and maximizing this objective function, we can obtain the optimal clustering results $C_{l}$ the corresponding the number of clusters $K_{l}$.
2.
$tr\left(U_{1}^{T}X_{1}^{T}X_{2}U_{2}\right)$ : to integrate shared clusters across datasets, we utilized CCA to project data $X_{1}$ and data $X_{2}$ into a common low-dimensional space, enabling the identification and propagation of shared information between two data. $U_{1}\in R^{N_{1}\times Q}$ and $U_{2}\in R^{N_{2}\times Q}$ are the Q-dimensional representations of data $X_{1}$ and data $X_{2}$, where Q is the number of canonical correlation vectors (CCVs).
3.
$\beta_{1}\sum_{k_{1}k_{2}}s_{k_{1}k_{2}}{\left(P\left(U_{1}^{T}C_{1}B_{1},U_{2}^{T}C_{2}B_{2}\right)+\beta_{2}J\left(\mathrm{DEG}\left(X_{1},C_{1}\right),\mathrm{DEG}\left(X_{2},C_{2}\right)\right)-\varepsilon \right)}_{k_{1}k_{2}}^{+}-\beta_{3}\sum_{k_{1}k_{2}}s_{k_{1}k_{2}}$ : we used the stable matching model (together with constraints) to calculate the cluster matching matrix S, where $s_{k_{1}k_{2}}$ is the value of $k_{1}$ rows and $k_{2}$ columns of S. $s_{k_{1}k_{2}}$ indicates whether the $k_{1}$-th cluster of data $X_{1}$ matches the $k_{2}$-th cluster of data $X_{2}$. If it matches, $s_{k_{1}k_{2}}=1$, otherwise $s_{k_{1}k_{2}}=0$. The function represents the correlation of matching pairs under $\lambda_{1}$ and $\lambda_{2}$. First, we defined two representations of clusters: A low-dimensional vector representation denoted by $U_{l}^{T}C_{l}B_{l}$ where $B_{l}=\mathrm{diag}(\frac{1}{b_{1}},\ldots ,\frac{1}{b_{K_{l}}})$, $b_{k_{l}}$ is the number of cells in the k-th cluster of the l-th data, and a set representation denoted by $\mathrm{DEG}\left(X_{l},C_{l}\right)$. The latter is calculated using the Wilcoxon rank sum test to identify differentially expressed genes within each cluster. Then we calculated the correlation between the clusters of data $X_{1}$ and data $X_{2}$ under these two representations. For the clusters in the vector representation, we calculated the correlation using the Pearson correlation coefficient:
  (2) $$\begin{array}{c} P\left(v_{k_{1}},v_{k_{2}}\right)=\frac{\sum_{q}\left(v_{qk_{1}}-{\bar{v}}_{k_{1}}\right)\left(v_{qk_{2}}-{\bar{v}}_{k_{2}}\right)}{\sqrt{\sum_{q}{\left(v_{qk_{1}}-{\bar{v}}_{k_{1}}\right)}^{2}\sum_{q}{\left(v_{qk_{2}}-{\bar{v}}_{k_{2}}\right)}^{2}}} \end{array}$$

where $v_{k_{l}}$ denoted the low-dimensional representation of the $k_{l}$-th cluster of the data $X_{l}$. For the clusters in the set representation, we calculated the correlation using the Jaccard coefficient:


(3) $$\begin{array}{c} J\left(e_{k_{1}},e_{k_{2}}\right)=\frac{\left|e_{k_{1}}\cap e_{k_{2}}\right|}{\left|e_{k_{1}}\cup e_{k_{2}}\right|} \end{array}$$

where $e_{k_{l}}$ denoted the set representation of the $k_{l}$-th cluster of data $X_{l}$. Then we combined two correlations and match the clusters of data $X_{1}$ and data $X_{2}$ using the stable matching algorithm. $\beta_{3}\sum_{k_{1}k_{2}}s_{k_{1}k_{2}}$ constrains the number of clusters, which determines the value of $\lambda_{l}$.

In the constraints, ${\left\|u_{l,j}\right\|}_{2}^{2}\leq 1$ is imposed to normalize the column vectors $u_{l,j}$ of $U_{l}$ using the 2-norm. $\sum_{k_{2}=1}^{K_{2}}s_{k_{1}k_{2}}\leq t\;$and $\sum_{k_{1}=1}^{K_{1}}s_{k_{1}k_{2}}\leq t$ constrain the clusters of each data to be matched with at most t clusters of another data. $t^{2}s_{k_{1}k_{2}}+t\sum_{j\in J_{k_{1}}}s_{k_{1}j}+\sum_{i\in I_{k_{2}}}s_{ik_{2}}\geq t^{2}$ ensures that the matching S is a stable match. $J_{k_{1}}$ and $I_{k_{2}}$ are defined as follows:


(4) $$\begin{array}{c} J_{k_{1}}≔\left\{j\in K_{2}|s_{k_{1}j}>s_{k_{1}k_{2}}\right\}\; \end{array}$$

(5) $$\begin{array}{c} I_{k_{2}}≔\left\{i\in K_{1}|s_{ik_{2}}>s_{k_{1}k_{2}}\right\}.\; \end{array}$$

### 2.3 Optimization algorithm

We proposed a heuristic iterative algorithm utilizing variable alternation to solve the nonconvex, nonlinear and mixed integer optimization problem (1) by dividing it into four sub-problems.

**Step 1:** Initialize $\lambda_{1}=\lambda_{10}$, $\lambda_{2}=\lambda_{20}$ and solve for the variables $C_{1}$, $C_{2}$, $U_{1}$, $U_{2}$;

**1.1:** Sub-problem 1:


(6) $$\begin{array}{c} \underset{C_{1}}{\max}\frac{1}{2m_{1}}\sum_{ij}\sum_{k_{1}}\left(a_{1,ij}-\lambda_{1}\frac{d_{1,i}d_{1,j}}{2m_{1}}\right)c_{1,ik_{1}}c_{1,jk_{1}}\\ s.t.\;c_{1,ik_{1}}=0,1;\;i=1,2,\ldots ,N_{1};\;k_{1}=1,2,\ldots K_{1} \end{array}$$

sub-problem 2:


(7) $$\begin{array}{c} \underset{C_{2}}{\max}\frac{1}{2m_{2}}\sum_{ij}\sum_{k_{2}}\left(a_{2,ij}-\lambda_{2}\frac{d_{2,i}d_{2,j}}{2m_{2}}\right)c_{2,ik_{2}}c_{2,jk_{2}}\\ s.t.\;c_{2,ik_{2}}=0,1;\;i=1,2,\ldots ,N_{2};\;k_{2}=1,2,\ldots K_{2} \end{array}$$

solving $C_{1}$, $C_{2}$ using the Louvain clustering algorithm for sub-problem 1 and sub-problem 2.

**1.2:** Sub-problem 3:


(8) $$\begin{array}{c} \underset{U_{1},U_{2}}{\max}tr\left(U_{1}^{T}X_{1}^{T}X_{2}U_{2}\right)\\ s.t.\;{u_{l,j}}_{2}^{2}\leq 1;\;j=1,2,\ldots ,N_{l};\;l=1,2 \end{array}$$

solving $U_{1}$, $U_{2}$ using singular value decomposition (SVD) for sub-problem 3. For computational efficiency, we use the “RunCCA” function from Seurat V3 to solve this problem.

**Step 2:** Fixing variables $C_{1}$, $C_{2}$, $U_{1}$, $U_{2}$ calculated by **Step 1** and solving for the alignment variable S;

**2.1:** Calculating two representations of the clusters, $U_{l}^{T}C_{l}B_{l}$ and $\mathrm{DEG}\left(X_{l},C_{l}\right)$, l=1,2;

**2.2:** Sub-problem 4:


(9) $$\begin{array}{l} \underset{S}{\max}\sum_{k_{1}k_{2}}s_{k_{1}k_{2}}(P\left(U_{1}^{T}C_{1}B_{1},U_{2}^{T}C_{2}B_{2}\right)\\ {+\beta_{2}J\left(\mathrm{DEG}\left(X_{1},C_{1}\right),\mathrm{DEG}\left(X_{2},C_{2}\right)\right)-\varepsilon )}_{k_{1}k_{2}}^{+}\\ -\beta_{3}\sum_{k_{1}k_{2}}s_{k_{1}k_{2}}\\ s.t.\;\sum_{k_{2}=1}^{K_{2}}s_{k_{1}k_{2}}\leq t;\;k_{1}=1,2,\ldots ,K_{1}\\ \sum_{k_{1}=1}^{K_{1}}s_{k_{1}k_{2}}\leq t;k_{2}=1,2,\ldots ,K_{2}\\ \;t^{2}s_{k_{1}k_{2}}+t\sum_{j\in J_{k_{1}}}s_{k_{1}j}+\sum_{i\in I_{k_{2}}}s_{ik_{2}}\geq t^{2};\\ \;k_{1}=1,2,\ldots ,K_{1};\;k_{2}=1,2,\ldots ,K_{2}\\ s_{k_{1}k_{2}}=0,\;1;\;k_{1}=1,2,\ldots ,K_{1};\;k_{2}=1,2,\ldots ,K_{2} \end{array}$$

sub-problem 4 is a 0–1 integer programming problem and we use the heuristic algorithm MNN (mutual nearest neighbors) (Stuart *et al.* 2019) or Gale–Shapley algorithm (Gale and Shapley 1962, Klein 2018) to compute the matching matrix S.

**Step 3:** Calculating the objective function value such that $\lambda_{10}=\lambda_{10}+h$ and $\lambda_{20}=\lambda_{20}+h$ as the initial values for the next round of iterations, where h is the step size, and repeat **Step 1**, **Step 2** and **Step 3** for n iterations.

**Step 4:** Output results; output the optimal solution after n iterations until that the value of the objective function is nonincreasing: ($\lambda_{1}$, $\lambda_{2}$, $C_{1}$, $C_{2}$, $U_{1}$, $U_{2}$, S).

In the optimization algorithm, when solving sub-problem 4, we set MNN as the default algorithm. In addition, when t=1, we also utilize the Gale–Shapley algorithm. In Supplementary Fig. S5c, we compare the results of these two algorithms using human and mouse pancreas data. We observed that the Gale–Shapley algorithm produced five additional matches (highlighted in yellow) compared to the MNN algorithm. However, the correlations for these additional matches were zero. This occurred because the Gale–Shapley algorithm attempts to match each cluster from the mouse data (D2) with the most correlated cluster in the human data (D1). If a match is rejected, the algorithm proceeds to the next most correlated cluster and repeats this process until all clusters in D2 are matched, even if the correlations are zero. While the MNN algorithm is heuristic and may not always provide the optimal solution for more complex problems, it effectively captures the most biologically relevant cluster matches between different species.

### 2.4 Parameter selection

There were four hyperparameters in our model: ε, $\beta_{1}$, $\beta_{2}$, $\beta_{3}$. ε represented the random noise in the paired clusters. $\beta_{1}$ denoted the weight coefficient of sub-problem 4, while $\beta_{2}$ denoted the weight coefficient of the two types of correlations. $\beta_{3}$ constrained $\lambda_{1}$ and $\lambda_{2}$ from taking too large a value by the average correlation of the paired clusters. Considering the slow computation speed of DEG, we used different hyper-parameter settings when optimizing $\lambda_{1}$ and $\lambda_{2}$ in n iterations than when optimizing $C_{1}$, $C_{2}$, $U_{1}$, $U_{2}$ and S using the optimal $\lambda_{1}$ and $\lambda_{2}$ in order to speed up the computation speed of the model.

When solving optimal $\lambda_{1}$ and $\lambda_{2}$ in n iterations, for different $\lambda_{1}$ and $\lambda_{2}$, we first solved sub-problem 1, sub-problem 2, and sub-problem 3, and then set


(10) $$\begin{array}{c} \varepsilon =P\left(\frac{\sum_{i=1}^{N_{1}}{{(U}_{1}^{T})}_{.i}}{N_{1}},\frac{\sum_{j=1}^{N_{2}}{{(U}_{2}^{T})}_{.j}}{N_{2}}\right) \end{array}$$

(11) $$\begin{array}{c} \beta_{1}=60 \end{array}$$

(12) $$\begin{array}{c} \beta_{2}=0 \end{array}$$

Next, solve the optimization problem


$$\begin{array}{l} \underset{S}{\max}\sum_{k_{1}k_{2}}s_{k_{1}k_{2}}m_{k_{1}k_{2}}\\ s.t.\;\sum_{k_{2}=1}^{K_{2}}s_{k_{1}k_{2}}\leq t;\;k_{1}=1,2,\ldots ,K_{1};\\ \sum_{k_{1}=1}^{K_{1}}s_{k_{1}k_{2}}\leq t;k_{2}=1,2,\ldots ,K_{2} \end{array}$$

(13) $$\begin{array}{l} \;t^{2}s_{k_{1}k_{2}}+t\sum_{j\in J_{k_{1}}}s_{k_{1}j}+\sum_{i\in I_{k_{2}}}s_{ik_{2}}\geq t^{2};\;\\ k_{1}=1,2,\ldots ,K_{1};\;k_{2}=1,2,\ldots ,K_{2}\\ s_{k_{1}k_{2}}=0,\;1;\;k_{1}=1,2,\ldots ,K_{1};\;k_{2}=1,2,\ldots ,K_{2} \end{array}$$

Where


(14) $$m_{k_{1}k_{2}}={\left(P\left(U_{1}^{T}C_{1}B_{1},U_{2}^{T}C_{2}B_{2}\right)+\beta_{2}J\left(\mathrm{DEG}\left(X_{1},C_{1}\right),\mathrm{DEG}\left(X_{2},C_{2}\right)\right)-\varepsilon \right)}_{k_{1}k_{2}}^{+}$$

Use the optimal S to set


(15) $$\begin{array}{c} \beta_{3}=\beta_{2}\frac{\left(\sum_{k_{1}k_{2}}s_{k_{1}k_{2}}-1\right)\sum_{k_{1}k_{2}}s_{k_{1}k_{2}}m_{k_{1}k_{2}}}{({\sum_{k_{1}k_{2}}s_{k_{1}k_{2}})}^{2}} \end{array}$$

When optimizing $C_{1}$, $C_{2}$, $U_{1}$, $U_{2}$ and S using the optimal $\lambda_{1}$ and $\lambda_{2}$, we first solved sub-problem 1, sub-problem 2, and sub-problem 3, and then set


(16) $$\begin{array}{c} \varepsilon =P\left(\frac{\sum_{i=1}^{N_{1}}{{(U}_{1}^{T})}_{.i}}{N_{1}},\frac{\sum_{j=1}^{N_{2}}{{(U}_{2}^{T})}_{.j}}{N_{2}}\right) \end{array}$$

(17) $$\begin{array}{c} \beta_{1}=60 \end{array}$$

(18) $$\begin{array}{c} \beta_{2}=\frac{\max P\left(U_{1}^{T}C_{1}B_{1},U_{2}^{T}C_{2}B_{2}\right)}{\max J\left(\mathrm{DEG}\left(X_{1},C_{1}\right),\mathrm{DEG}\left(X_{2},C_{2}\right)\right)} \end{array}$$

(19) $$\begin{array}{c} \beta_{3}=0 \end{array}$$

where the max function select the maximum value from a set of values.

Notably, when solving for the optimal $\lambda_{1}$ and $\lambda_{2}$, we set $\beta_{2}=0$ [Equation (12)]. This approach is intended to accelerate the computation, as calculating DEGs for every combination of $\lambda_{1}$ and $\lambda_{2}$ would be computationally intensive and time-consuming. Once the optimal $\lambda_{1}$ and $\lambda_{2}$ are determined, we then set $\beta_{2}$ as Equation (18). The reason is to balance the influence of CCA and DEGs on the matching process, thereby mitigating the risk of over correction caused by CCA.

### 2.5 Hyperparameters sensitivity analysis

we have incorporated experimental analyses to demonstrate the sensitivity of ClusterMatch to different settings of hyperparameters, specifically ε, $\beta_{2}$ and *t* (Supplementary Fig. S5d). We evaluate the sensitivity of these three hyperparameters in the integration scenario, using the *F*1 score based on the batch and cell type silhouette coefficients as the evaluation metric. For the sensitivity analysis of $\beta_{2}$, we use the human dendritic datasets, while for the ε and t, we utilize the PBMC datasets.

As shown in Supplementary Fig. S5d, for the sensitivity analysis of $\beta_{2}$, *F*1 score values range from 0.5123 to 0.5902 with median of 0.5407 and a standard deviation of 0.0249. It is noteworthy that the default value of $\beta_{2}=1.85$ corresponded to the maximum *F*1 score within this range. For the sensitivity analysis of ε, *F*1 score values range from 0.5267 to 0.5342 with median of 0.5306 and a standard deviation of 0.0029. For the sensitivity analysis of *t*, *F*1 score values range from 0.4917 to 0.5006 with median of 0.4989 and a standard deviation of 0.0033. These results indicate that ClusterMatch exhibits robustness to variations in ε, $\beta_{2}$, and *t* during the alignment and integration of datasets.

### 2.6 Updating the low-dimensional representation of cells

To reduce the effect of overcorrection of CCA, we used the output from ClusterMatch that contained both local and global information to update the cell embeddings. This approach ensures that the low-dimensional representations of matched clusters become identical, facilitating the convergence of cells within these clusters. The update steps are as follows.

1. Update the cluster embeddings $V_{1}=U_{1}^{T}C_{1}B_{1}$ and $V_{2}=U_{2}^{T}C_{2}B_{2}$ based on the matching result S:
  (20) $$\begin{array}{c} V_{1}^{\prime }=V_{1}+\alpha V_{2}{\left(S-J\right)}^{T} \end{array}$$
   
  (21) $$\begin{array}{c} V_{2}^{\prime }=V_{2}+\alpha V_{1}\left(S-J\right) \end{array}$$
   
  (22) $$\begin{array}{c} V_{1}^{\prime \prime }=V_{1}^{\prime }+V_{2}^{\prime }S^{T} \end{array}$$
   
  (23) $$\begin{array}{c} V_{2}^{\prime \prime }=V_{2}^{\prime }+V_{1}^{\prime }S \end{array}$$
  where α is the parameter that regulates the distance and J is a $K_{1}\times K_{2}$ dimensional matrix with all elements of 1.
2. Update the cell embeddings $U_{1}$ and $U_{2}$ based on $V_{1}^{\prime \prime }$ and $V_{2}^{\prime \prime }$:
  (24) $$\begin{array}{c} U_{1}^{\prime }=U_{1}+C_{1}{\left(V_{1}^{\prime \prime }-V_{1}\right)}^{T} \end{array}$$
   
  (25) $$\begin{array}{c} U_{2}^{\prime }=U_{2}+C_{2}{\left(V_{2}^{\prime \prime }-V_{2}\right)}^{T} \end{array}$$
3. Update the cell embeddings based on the correlation of cells and clusters:
  (26) $$\begin{array}{c} E=U^{T}V \end{array}$$

Where $U=L_{2}\left({U_{1}^{\prime }}^{T},{U_{2}^{\prime }}^{T}\right)\;$and $V=L_{2}\left(V_{1}^{\prime \prime },V_{2}^{\prime \prime }\right)$, $L_{2}$ was $L_{2}$ normalization


(27) $$\begin{array}{c} L_{2}\left(x_{1},x_{2},\;\ldots x_{n}\;\right)=\left(\frac{x_{1}}{{\left\|x_{1}\right\|}_{2}},\;\frac{x_{2}}{{\left\|x_{2}\right\|}_{2}},\;\ldots \frac{x_{n}}{{\left\|x_{n}\right\|}_{2}}\right) \end{array}$$

### 2.7 Identification of marker genes in mutually most correlated clusters

ClusterMatch utilized the “FindAllMarkers” function from Seurat V3 to identify differentially expressed genes within each cluster of the reference and query datasets using the Wilcoxon rank sum test. The overlapping set of differentially expressed genes between the mutually most correlated clusters in the reference and query datasets is considered as the set of differentially expressed genes for the mutually most correlated clusters.

### 2.8 Cell type annotations based on reference data

ClusterMatch supported annotating cell types in the query dataset lacking annotations, by utilizing the high-quality annotations of the reference dataset. Supposing $X_{1}\in R^{G\times N_{1}}$ and $X_{2}\in R^{G\times N_{2}}$ were scaled scRNA-seq matrices for the reference and query datasets, and $C_{1}\in {\left\{0,1\right\}}^{N_{1}\times K_{1}}$ was the cell type annotations for the reference dataset. First, we used Louvain clustering algorithm (default resolution $\lambda_{2}=2$) to solve for the initial clustering result $C_{2}\in {\left\{0,1\right\}}^{N_{2}\times K_{2}}$ of $X_{2}$. Second, we used CCA to reduce the dimensionality of $X_{1}$ and $X_{2}$, obtaining their low dimensional representations $U_{1}\in R^{N_{1}\times Q}$ and $U_{2}\in R^{N_{2}\times Q}$. Third, we used a matching algorithm to match the clusters of the query dataset with the reference data cluster with the highest correlation


(28) $$\begin{array}{c} \underset{S}{\max}\sum_{k_{1}k_{2}}s_{k_{1}k_{2}}{\left(P\left(U_{1}^{T}C_{1}B_{1},U_{2}^{T}C_{2}B_{2}\right)+\beta_{2}J\left(\mathrm{DEG}\left(X_{1},C_{1}\right),\mathrm{DEG}\left(X_{2},C_{2}\right)\right)-\varepsilon \right)}_{k_{1}k_{2}}^{+}\\ \begin{array}{c} s.t.\;\sum_{k_{1}=1}^{K_{1}}s_{k_{1}k_{2}}\leq 1;\;s_{k_{1}k_{2}}=0,\;1;\;k_{2}=1,2,\ldots ,K_{2} \end{array} \end{array}$$

Where ε and $\beta_{2}$ were computed according to Equations (16) and (18). Finally, we annotated the query data using the cell type annotations of the reference data


(29) $$\begin{array}{c} C_{2}^{\prime }=C_{2}S^{T} \end{array}$$

Where $C_{2}^{\prime }\in {\left\{0,1\right\}}^{N_{2}\times K_{1}}$ was the cell type annotations of query.

### 2.9 Alignment process of cattle and sheep data

In the process of aligning sheep and cattle data using the ClusterMatch method, we first align the sheep’s F30 data with the F90 data, then align the F90 data with the F130 data, then align the F130 data with the B45 data, and finally align the B45 data with the cattle’s rumen data.

### 2.10 Evaluation metrics

#### 2.10.1 Silhouette coefficient

To evaluate the effectiveness of integrating embeddings using different methods, we calculate the silhouette coefficient for each cell by considering two different groups: (i) grouping based on the batches, called the batch silhouette coefficient ($s_{\mathrm{batch}}$); (ii) grouping based on annotated cell types, called the cell type silhouette coefficient ($s_{\mathrm{celltype}}$). The different methods used various types of embedding spaces to compute the Euclidean distance. Then we summarized the two silhouette coefficients by calculating an *F*1 score as follows:


(30) $$\begin{array}{c} {F1}_{\mathrm{sil}}=\frac{2\cdot \left(1-s_{\mathrm{batch}}^{\prime }\right)\cdot s_{\mathrm{celltype}}^{\prime }}{1-s_{\mathrm{batch}}^{\prime }+s_{\mathrm{celltype}}^{\prime }} \end{array}$$

where $s^{\prime }=\left(s+1\right)/2$. A higher *F*1 score indicates better performance in the alignment of the batches as well as the preservation of biological signals.

#### 2.10.2 Cell type annotation evaluation metrics

To evaluate the accuracy of cell type annotation, we used precision, recall, and *F*1 score as three metrics for assessment. We calculated the precision, recall, and *F*1 score for all cell types and obtained their average values. Specifically, the precision, recall, and *F*1 score for each cell type were determined by computing the true positive (TP), false positive (FP), true negative (TN), and false negative (FN) for that particular cell type.


(31) $$\begin{array}{c} \mathrm{Precision}=\frac{TP}{TP+FP},\;\mathrm{Recall}=\frac{TP}{TP+FN}\;\\ \;{F1}_{\mathrm{annotation}}=\frac{2\cdot \mathrm{Precision}\cdot \;\mathrm{Recall}}{\mathrm{Precision}+\mathrm{Recall}} \end{array}$$

#### 2.10.3 Adjusted rand index

We utilized the Adjusted Rand Index (ARI) as a metric to evaluate the clustering of cell types. The ARI is defined as follows:


(32) $$\begin{array}{c} \mathrm{ARI}=\frac{RI-E\left(RI\right)}{\max\left(RI\right)-E\left(RI\right)}\; \end{array}$$

Here, $E\left(\cdot \right)$ is the expectation and RI is the Rand Index which is defined as follows:


(33) $$\begin{array}{c} RI=\frac{TP+TN}{TP+FP+FN+TN} \end{array}$$

### 2.11 Data availability

We evaluated the alignment performance of ClusterMatch on the following scRNA-seq datasets. Here are the specific descriptions of each dataset, including data sources, data batches, cell counts, and sequencing method:

#### 2.11.1 Dataset 1: human pancreas dataset

We downloaded the processed gene expression matrix and cell type annotation of human pancreas scRNA-seq data from the GEO accession GSE84133. The data was obtained using the inDrops sequencing method. In order to ensure the reliability of the data, we specifically selected cell types with a cell number >30 as our test data. Our test data consists of 8506 cells, including 10 annotated cell types, namely acinar, activated stellate, alpha, beta, delta, ductal, endothelial, gamma, macrophage, and quiescent stellate cells.

#### 2.11.2 Dataset 2: mouse pancreas dataset

The scRNA-seq data for the mouse pancreas was also obtained from GEO accession GSE84133, following the same processing steps as the human pancreas data. For the test data, we also selected cell types with cell counts >30. The resulting dataset comprises 1805 cells, including seven annotated cell types: Alpha, beta, delta, ductal, endothelial, gamma, and quiescent stellate cells.

#### 2.11.3 Dataset 3: human dendritic cells dataset

The scRNA-seq dataset for human dendritic cells was obtained from the GEO accession GSE80171. This dataset consists of two batches of scRNA-seq data that were sequenced using the Smart-Seq2 (Villani *et al.* 2017). Four distinct cell populations were identified in this dataset: CD1C DC, CD141 DC, plasmacytoid DC, and double-negative cells. To ensure nonoverlapping cell types between the batches, Tran *et al.* removed the CD1C DC cell type from batch 1 and the CD141 DC cell type from batch 2. As a result, batch 1 consists of 288 cells with three annotated cell types: CD141 DC, double-negative, and plasmacytoid DC. Similarly, batch 2 also contains 288 cells with three annotated cell types: CD1C DC, double-negative, and plasmacytoid DC. The two batches share two common cell types, namely plasmacytoid DC and double-negative, with one unshared cell type in each batch (CD141 DC in batch 1 and CD1C DC in batch 2).

#### 2.11.4 Dataset 4: PBMC dataset

We obtained the processed gene expression matrix and cell type annotation of PBMC scRNA-seq data from the SeuratData package https://github.com/satijalab/seurat-data (pbmcsca v3.0.0) (Ding *et al.* 2019). The dataset consists of two batches of PBMC data, generated using 10× Genomics method and inDrops method. The 10× Genomics batch comprises 3222 cells, while the inDrops batch contains 6584 cells. Each batch of data includes nine distinct cell types: CD14+ monocyte, dendritic cell, cytotoxic T cell, CD16+ monocyte, plasmacytoid dendritic cell, B cell, natural killer cell, CD4+ T cell, and megakaryocyte.

#### 2.11.5 Dataset 5: human and macaque dLGN dataset

We obtained the processed dLGN data for the human and macaque from the ALLEN Brain Map https://portal.brain-map.org/atlases-and-data/rnaseq/comparative-lgn. The human dLGN dataset consists of 952 cells, while the macaque dLGN dataset consists of 1723 cells. The data for both species were sequenced using the Smart-Seq. The annotations were further refined through classification and manual review, resulting in four cell types: Koniocellular (K), magnocellular and parvocellular projection neurons (MP), and two GABAergic cells (GABA1 and GABA2). To ensure consistency across the datasets, we checked the original cell type annotations from both human and macaque data manually and reannotated the labels. In the macaque dataset, the magnocellular projection neurons (M) and parvocellular projection neurons (P) were collectively referred to as MP. The GABAergic cells labeled GABA3 and GABA4 were collectively referred to as GABA2. In the human dataset, the GABAergic cells labeled GABA3 were also referred to as GABA2.

#### 2.11.6 Dataset 6: cattle rumen

The scRNA-seq dataset for cattle rumen cells was obtained from the GEO accession GSE176512, which utilized the 10× Genomics method for sequencing. A total of 25 828 cells were obtained from the cattle rumen dataset and it was annotated into 11 distinct cell types.

#### 2.11.7 Dataset 7: sheep rumen

The scRNA-seq dataset for sheep rumen cells was obtained from the Genome Sequence Archive database accession CRA007511, which utilized the 10× Genomics method for sequencing. From the sheep dataset, we specifically selected four developmental time points: 30 days of gestation (F30), 90 days of gestation (F90), 130 days of gestation (F130), and 45-day-old lambs (B45). The corresponding cell counts at these time points were 5010, 7595, 6760, and 17 882, respectively. The sheep rumen dataset was annotated into 12 distinct cell types.

### 2.12 Translating features cross-species by gene homology

We utilized Ensembl’s BioMart to convert orthologous genes. Specifically, we used the following genome assemblies: Human (GRCh38), mouse (GRCm39), macaque (Mmul_10), cattle (ARS-UCD1.3), and sheep (ARS-UI_Ramb). For the homology type, we selected ortholog_one2one.

## 3 Results

### 3.1 Overview of ClusterMatch

We developed ClusterMatch to align cell clusters, which, according to Waddington’s concept of the epigenetic landscape, are metaphorically called as attractor states in dynamical systems and exhibit robustness to stochastic fluctuations (Bhattacharya *et al.* 2011, Ferrell 2012, Li *et al.* 2016, Waddington 2014) and are expected to offer enhanced stability and reliability compared to individual cell-level alignment. In addition, ClusterMatch attacks the intrinsic multi-scale difficulty in widely used heuristic clustering algorithm for high-dimensional and high-noise scRNA-seq data (Luecken and Theis 2019). Technically, ClusterMatch is a unified multi-objective optimization model that identify clusters, represent clusters, and align clusters in scRNA-seq data.

The input of our ClusterMatch model is two scRNA-seq data $X_{1}$ and $X_{2}$, and the output is the alignment graph at the cluster level (Fig. 1a). The delineation and number of clusters are, as well as the alignment, achieved through the optimization of the model [Equation (1)]. There are three major components: Multi-scale clustering with optimized resolution, dimensional reduction by CCA, and stable matching. The objective function is constructed in Fig. 1b. The first and second clustering terms derive the clustering of two scRNA-seq data by maximizing multi-scale modularity (Traag *et al.* 2019), resulting in clustering matrices $C_{1}$ and $C_{2}$ for each dataset. $\lambda_{1}$ and $\lambda_{2}$ are clustering resolutions to be optimized. The third term is the CCA term that projects two scRNA-seq data into a shared low-dimensional space represented by $U_{1}$ and $U_{2}$. Thus, molecular patterns in both datasets that share the same biological meaning can be captured and represented uniformly in a low dimensional space (Song *et al.* 2021). Integrating the first, the second and the third terms, we define two representations of clusters; one is the dimensional vector representation $U_{1}^{T}C_{1}B_{1}$ and $U_{2}^{T}C_{2}B_{2}$ which is the mean vector of all cells in each cluster, where $B_{l}=\mathrm{diag}(\frac{1}{b_{1}},\ldots ,\frac{1}{b_{K_{l}}})$ and $b_{k_{l}}$ is the number of cells in the k-th cluster of the l-th data. The other is the set representation of differentially expressed genes $\mathrm{DEG}\left(X_{1},C_{1}\right)$ and $\mathrm{DEG}\left(X_{2},C_{2}\right)$. The correlation between the clusters under the two representations was then calculated using the Pearson correlation coefficient and the Jaccard coefficient. The fourth and fifth objective function terms, together with the constraints referred to as stable matching terms, match similar or identical clusters at different resolutions based on the correlation of the two datasets, where *S* is the matching result and used for constructing the output alignment graph. The formal optimization model is illustrated in Fig. 1b.

The ClusterMatch model utilizes both the global and local information when representing and aligning clusters of the two datasets. Specifically, in representing clusters, the vector representation incorporates the global information integrated by CCA for the two data and merges the local information separately clustered for each data. Moreover, the set representation fully leverages the local information clustered for each dataset and represents clusters from a saliency perspective, effectively avoiding the over-correction problem caused by insufficient consideration of local information in CCA (Haghverdi *et al.* 2018, Luecken *et al.* 2022). The details of the input, output, and modeling of ClusterMatch can be found in Section 2.

### 3.2 ClusterMatch identifies cell clusters mutually corroborating each other

To assess the efficacy of the alignment results obtained through ClusterMatch, we performed an analysis using scRNA-seq data acquired from human and mouse pancreas tissues, as previously reported by Baron *et al.* (Baron *et al.* 2016). Our analysis involved a comprehensive comparison of the alignment results with the manually annotated cell types provided by Baron *et al.* (Baron *et al.* 2016), as well as the alignment results achieved at different clustering resolutions.

**Figure 2.**
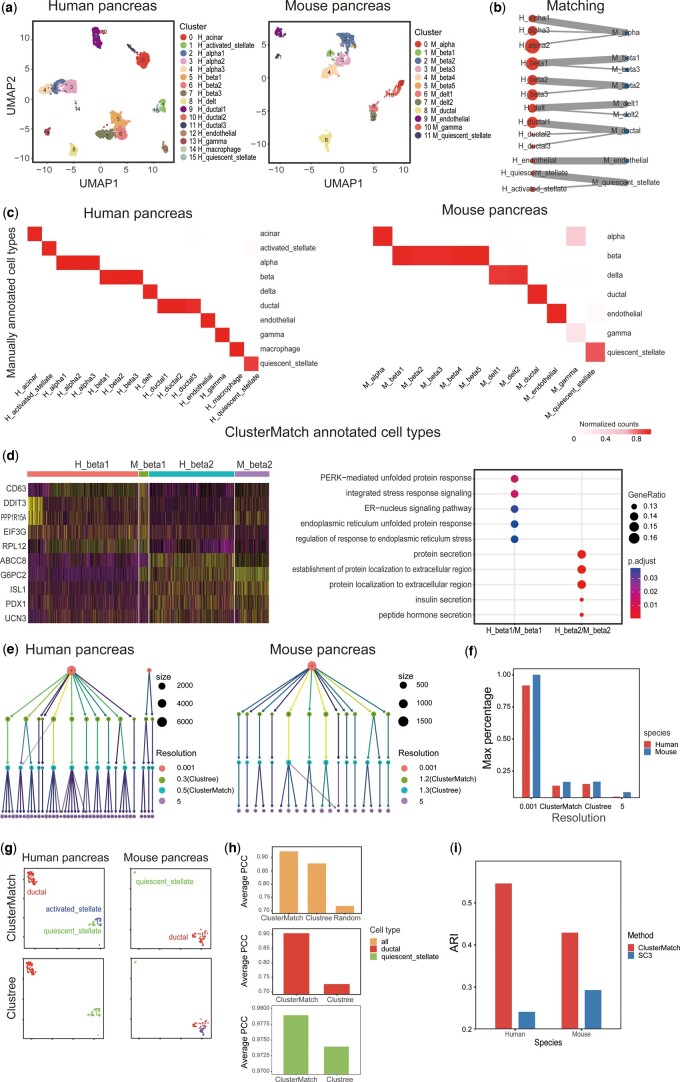
Validation of ClusterMatch by alignment of human and mouse single cell pancreas data. (**a**) UMAP visualization of human and mouse pancreas data, colored by ClusterMatch results. (**b**) ClusterMatch alignment of human and mouse pancreas clusters. The thickness of correspondence edges denotes confidence. (**c**) The fractions of agreement between annotated cell types by ClusterMatch and the manually annotated cell types given in Baron *et al.* (**d**) Heatmap of marker gene expression (left) and GO enrichment analysis (right) for two beta cell subtypes in human and mouse. (**e**) Clustree plots of human and mouse pancreas data showing the size and the number of clusters for different values of the clustering resolution parameter. (**f**) Proportion of cells in the cluster with the highest number of cells at different resolutions. (**g**) UMAP visualization of ductal and stellate cells from human and mouse pancreas, grouped by ClusterMatch and Clustree. (**h**) Average Pearson correlation coefficient between ClusterMatch and Clustree in all mutually most correlated clusters, Random indicates the correlation of all cells between human and mouse pancreas data (top). Average Pearson correlation coefficient between ClusterMatch and Clustree in ductal cells with mutually most correlated clusters (middle). Average Pearson correlation coefficient between ClusterMatch and Clustree in stellate cells with mutually most correlated clusters (bottom). (**i**) Bar plot of ARI for ClusterMatch and SC3 for human and mouse pancreas data

We first evaluated the alignment results of ClusterMatch using manually annotated cell types. Through multi-scale clustering and the alignment of scRNA-seq datasets, we finally classified human pancreatic cells into 16 clusters and mouse pancreatic cells into 11 clusters (Fig. 2a, Supplementary Fig. S1a). Moreover, ClusterMatch identifies seven mutually most correlated clusters (bold line) and eight mutually correlated clusters between the two species (Fig. 2b). The seven mutually most correlated clusters correspond to the main cell types found in the pancreas, namely: Alpha cells, beta cells (including two subtypes), delta cells, ductal cells, endothelial cells, and stellate cells. Significantly, ClusterMatch also provides a list of marker genes that are shared among the mutually most correlated clusters (Supplementary Fig. S1b), thereby enabling further functional characterization of these clusters and enhancing model interpretability. For example, specific marker genes such as TTR and IRX2 are associated with alpha cells, INS and IAPP with beta cells, SST with delta cells, KRT19 with ductal cells, and PLVAP and FLT1 with endothelial cells. Importantly, all of these marker genes can be validated and confirmed in the CellMarker2 database (Hu *et al.* 2023). Furthermore, Supplementary Fig. S1c revealed a lower number of marker genes compared to those identified by Seurat V3 using a single dataset, but with higher accuracy.

Moreover, clusters identified by ClusterMatch are consistent with those derived from manual annotation. For example, in both human and mouse pancreas datasets, ClusterMatch accurately identifies and annotates over 95% of the alpha, beta, delta, and ductal cells at high resolution (Fig. 2c). These findings underscore the ability of ClusterMatch to cluster datasets at high resolution. ClusterMatch demonstrates higher resolution than manual annotation for cell types in human and mouse datasets. In human and mouse beta cells, we identified two mutual most correlated clusters by ClusterMatch. Further analysis by GO terms: One subtype pertains to insulin secretion, while the other is associated with endoplasmic reticulum (ER) stress function (Fig. 2d). Our results corroborate prior research suggesting that beta cells are under ER stress due to their high demand for insulin secretion, and that beta cells exhibiting active ER stress are more prone to proliferation (Marchetti *et al.* 2007, Sharma *et al.* 2015).

To deal with multi-scale in single cell clustering, widely used Seurat V3 adopted the Louvain and Leiden algorithms to maximize modularity. The number of clusters found relies on a single tuning parameter called the resolution. This parameter is typically selected by manual inspection of the clusters. ClusterMatch optimizes this resolution parameter by borrow information from each other scRNA-seq dataset and expects better identification of cell clusters. We then compared the clustering resolutions obtained by ClusterMatch to those obtained using two extreme resolutions (0.001 and 5) and the Clustree (Qadir *et al.* 2020, Sundell *et al.* 2023, Zappia and Oshlack 2018) method (Fig. 2e, Supplementary Fig. S1d). Extreme resolutions can lead to overly many or few clusters (Fig. 2e and f), hindering accurate cell type identification. In addition, we evaluated the average Pearson correlation coefficient (PCC) between mutual most correlated clusters produced by ClusterMatch and Clustree methods (Fig. 2g and h, Supplementary Fig. S1e and f) to assess clustering results. Notably, ClusterMatch exhibited a superior average PCC of 0.94 compared to Clustree’s average PCC of 0.87 and the random PCC of 0.72. This superiority arises from ClusterMatch’s effective utilization of information mutually between datasets for resolution selection, which is more reasonable than Clustree’s reliance solely on single dataset information. Specifically, we observed a higher PCC between mouse and human quiescent stellate cells compared to the PCC between mouse quiescent stellate cells and human stellate cells. Consequently, ClusterMatch successfully distinguished activated and quiescent stellate cells in humans based on insights drawn from mouse quiescent stellate cells, an achievement not accomplished by Clustree. Furthermore, in the case of mouse ductal cells, Clustree divided them into two clusters, whereas ClusterMatch did not make such a distinction. ClusterMatch achieved the highest PCC between mutually most correlated clusters for ductal cells, leading to clustering results that aligned more consistently with manual annotation. In addition, we used the ROGUE metric (Liu *et al.* 2020) to assess the clustering results of ClusterMatch, which quantifies the purity of cell clusters (Supplementary Fig. S1g). The results demonstrate that ClusterMatch achieves an average ROGUE score exceeding 0.7 for both human and mouse pancreas data. Notably, in the mouse data, ClusterMatch exhibits a local maximum in terms of average ROGUE score. Finally, we used the manual annotation of cell types as the “gold standard” and computed the Adjusted Rand Index (ARI) to measure the similarity between ClusterMatch, SC3 (Kiselev *et al.* 2017) clustering results, and manually annotated cell types. As depicted in Fig. 2i, ClusterMatch outperformed SC3 in terms of similarity. In summary, by utilizing mutual information between datasets, ClusterMatch provides more meaningful and reliable cell clustering and alignment results.

### 3.3 ClusterMatch removes batch effect and better integrates scRNA-seq datasets

To assess the efficacy of ClusterMatch in jointing global and local information within datasets, we analyzed and integrated two batches of human dendritic datasets (Tran *et al.* 2020). These datasets consisted of different types of human blood dendritic cells, including CD1C DC, CD141 DC, plasmacytoid DC (pDC), and double-negative cells. Tran *et al.* had previously processed and manually split the data into two batches. Batch 1 included 96 pDC, 96 double-negative cells, and 96 CD141 cells, whereas batch 2 included 96 pDC, 96 double-negative cells, and 96 CD1C cells. It’s important to note that CD141 cells were only present in batch 1, while CD1C cells were only present in batch 2.

**Figure 3.**
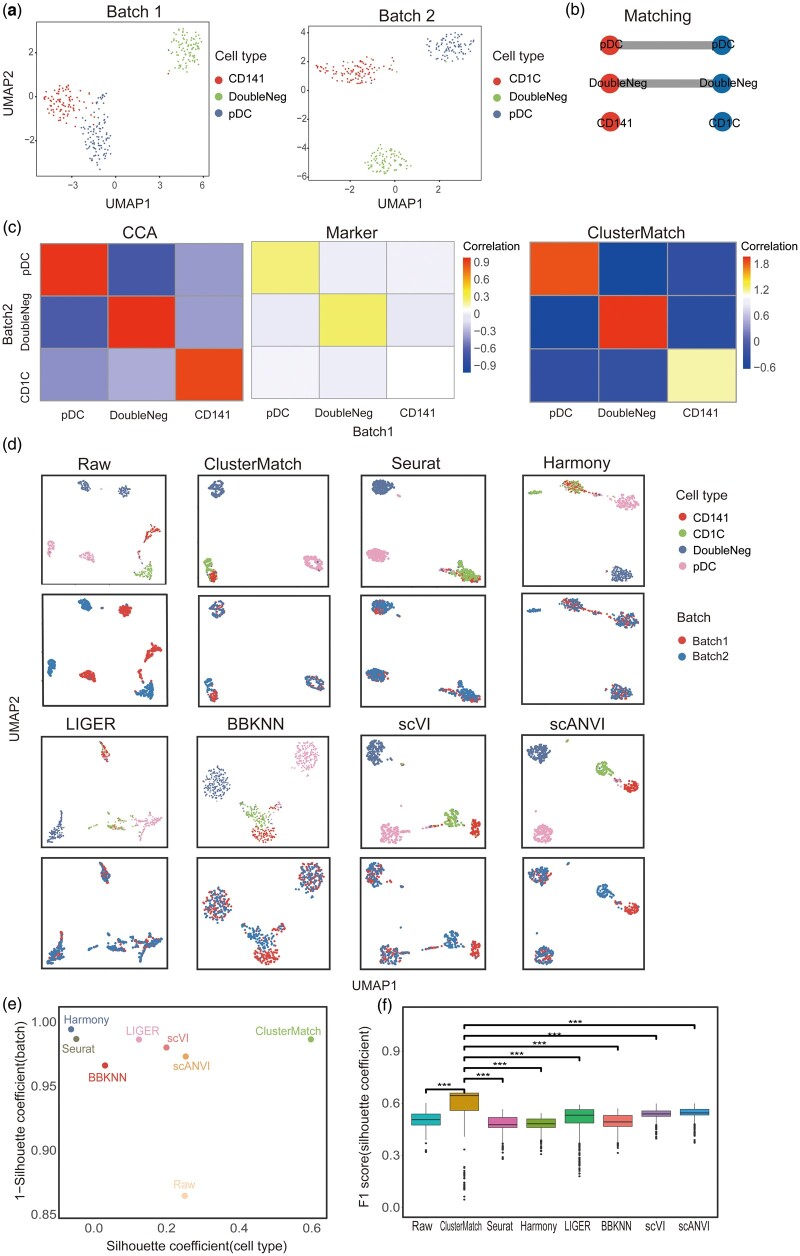
Integration of two batches of human dendritic cells. (**a**) UMAP visualization of two batches of human dendritic cells, colored by ClusterMatch. (**b**) ClusterMatch alignment of two batches clusters. (**c**) Heatmap of correlation between two batches in CCA vector representation, marker gene set representation and ClusterMatch model. (**d**) UMAP visualization of two integrated human dendritic datasets with nonoverlapping cell types by ClusterMatch, Seurat V3, Harmony, LIGER, BBKNN, scVI, and scANVI. The top panel is colored by the annotated cell types, and the bottom panel is colored by the datasets. (**e**) Scatter plot of mean silhouette coefficients for ClusterMatch, Seurat V3, Harmony, LIGER, BBKNN, scVI, and scANVI, where the *x* axis denotes the mean cell type silhouette coefficients, and the *y* axis denotes 1 minus mean batch silhouette coefficients; ideal outcomes would lie in the top right corner. ClusterMatch outperforms other four methods. (**f**) Boxplots of *F*1 scores of silhouette coefficients for ClusterMatch, Seurat V3, Harmony, LIGER, BBKNN, scVI, and scANVI. ClusterMatch significantly outperforms other four methods as determined by a t-test analysis (*P* < 0.001), denoted by ***

Jointing global and local information of datasets can accurately provide the cluster alignment. ClusterMatch accurately identified four cell types and matched them between the two batches of data (Fig. 3a and b, Supplementary Fig. S2a). To further explore the impact of cluster representations on cluster alignment, we examined the vector representation obtained through CCA. This approach allowed the correlation coefficients of the corresponding pDCs and double-negative cells in both batches to exceed 0.9. However, it also resulted in CD1C and CD141 correlations as high as 0.9 (Fig. 3c). To reduce the correlation between CD1C and CD141 which are different cell types, we explored the set representation of differentially expressed genes (DEGs), which contains more local information. By utilizing this approach, the correlation coefficient between CD141 and CD1C cells significantly dropped to only 0.07, much lower than that of the corresponding pDC and double-negative cells across the two batches. Therefore, we combined the correlations obtained from both representations, successfully matching the same cell types while avoiding the incorrect alignment of biologically distinct CD141 and CD1C cells.

To further illustrate the advantages of ClusterMatch in leveraging both global and local information, we applied the alignment results to integrate scRNA-seq data. Specifically, we integrated the two batches of dendritic cell data mentioned above and compared them with the methods Seurat V3, Harmony, LIGER, BBKNN, scVI and scANVI. Our findings revealed that Seurat V3, which performed integration at the cell level using CCA, clustered the CD141 and CD1C cells together, leading to overcorrection issues (Fig. 3d). ClusterMatch and LIGER performed integration at the cluster level and separated the CD141 and CD1C cells into two independent groups, while Harmony did not make such a distinction. The integration results were evaluated using the quantitative metric silhouette coefficient (Fig. 3e). ClusterMatch exhibited significantly higher cell type silhouette coefficients compared to all other methods, while maintaining similar batch silhouette coefficients. Overall, ClusterMatch achieved the highest median *F*1 score of silhouette coefficients, striking a better balance between removing batch variations and preserving cell type signals (Fig. 3f). In addition, we integrated peripheral blood mononuclear cell (PBMC) data from Indrop and 10X technologies (Supplementary Fig. S2b). Based on silhouette coefficients, ClusterMatch outperformed other methods in terms of cell type silhouette coefficients, batch silhouette coefficients, and median *F*1 score of silhouette coefficients (Supplementary Fig. S2c and d).

In summary, ClusterMatch effectively joints global and local information to align clusters, achieving a balance between batch removal and preservation of biological variation. Furthermore, it performs the integration of scRNA-seq data at the cluster level. This study highlights the significance of incorporating differentially expressed genes in the alignment process to mitigate overcorrection issues associated with CCA.

### 3.4 ClusterMatch improves cell type annotation accuracy by cluster alignment graph

ClusterMatch aims to align two scRNA-seq datasets at cluster level, which provides more interpretability and allows large scale dataset by saving computation time. In addition, the output cluster alignment graph improves cell annotation accuracy. We compared the accuracy of cluster alignment and cell alignment by applying ClusterMatch to annotate cell types in scRNA-seq data. To achieve this, we utilized scRNA-seq datasets from the dorsal lateral geniculate nucleus (dLGN) of both human and macaque brains (Bakken *et al.* 2021). The human dataset consisted of 952 cells, including four manually annotated cell types: Magnocellular and parvocellular neurons (MP), koniocellular neurons (K), and two types of GABAergic neurons (GABA1, GABA2). Similarly, the macaque dataset included 1724 cells, with the same four cell types as in humans.

**Figure 4.**
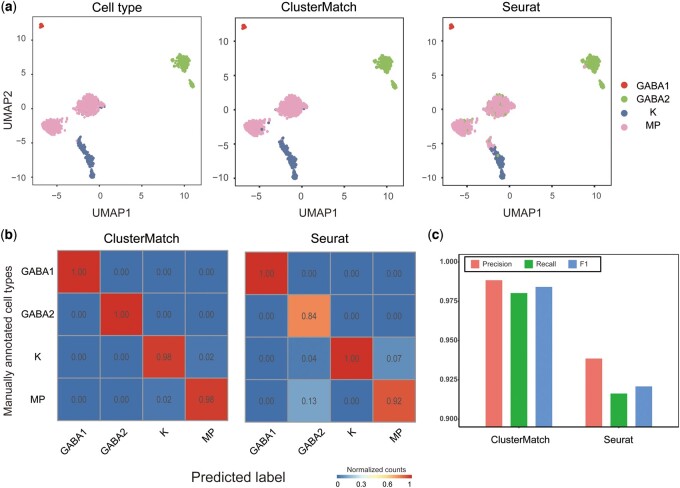
Annotation of macaque dLGN data using labeled human dLGN data. (**a**) UMAP visualization of macaque dLGN data, colored by manually annotated cell types given in Bakken *et al.*, ClusterMatch predicted labels, and Seurat V3 predicted labels. (**b**) Predicted labels by ClusterMatch and Seurat V3 and their fractions of agreement with the manually annotated cell types. (**c**) ClusterMatch and Seurat V3 cell type annotation performance on macaque dLGN data, evaluated by three classification metrics, precision, recall, and *F*1. ClusterMatch systematically outperforms Seurat V3

We used the human dLGN data as the reference data and the macaque dLGN data as the query data to annotate cell types in the query data, and used ClusterMatch to perform clustering and cluster alignment (Supplementary Fig. S3a and b). To compare the accuracy of cluster alignment with that of cell alignment, we analyzed Seurat V3 based on cell alignment against ClusterMatch, using manually annotated macaque cell types as the ground truth (Fig. 4a). Our analysis showed that ClusterMatch achieved over 98% accuracy in every cell type, whereas errors in Seurat V3 were concentrated mainly in GABA2 and MP cell types. Specifically, about 13% of Seurat V3 predicted GABA2 cells were misclassified as MP cells, while approximately 7% of predicted MP cells were K cells (Fig. 4b). This resulted in a lower *F*1 score for Seurat V3 (about 0.92) compared to ClusterMatch (about 0.98), as shown in Fig. 4c. Finally, we conducted an evaluation to assess the impact of using various resolution parameters for the query dataset on prediction accuracy (Supplementary Fig. S3c). Our findings indicated that the influence of different parameters on the results was negligible.

In general, we used the cluster alignment of ClusterMatch to help cell type annotation. Comparing with Seuart V3, it shows that the cluster alignment is more accurate and reliable than the cell alignment.

### 3.5 ClusterMatch reveals conserved cell types and differentiation diagram in rumen development

We finally apply ClusterMatch in cross-species developmental scRNA-seq data alignment and demonstrate its potential for biological insights. The rumen, as a significant evolutionary innovation and a unique organ, plays a vital role in the adaptation of ruminant animals to complex ecological environments (Pan *et al.* 2022). To explore the distinctive digestive and absorptive functions of the rumen, we used ClusterMatch on rumen data from sheep (Yuan *et al.* 2022) and cattle (Wu *et al.* 2022). We selected four developmental time points from the sheep dataset, including 30 days of gestation (F30), 90 days of gestation (F90), 130 days of gestation (F130), and 45-day-old lambs (B45). For cattle rumen scRNA-seq data, we utilized the adult cattle. We analyzed a total of 63 075 cells from the rumen scRNA-seq data. The corresponding cell counts for sheep at F30, F90, F130, B45, and cattle were 5010, 7595, 6760, 17 882, and 25 828, respectively.

**Figure 5.**
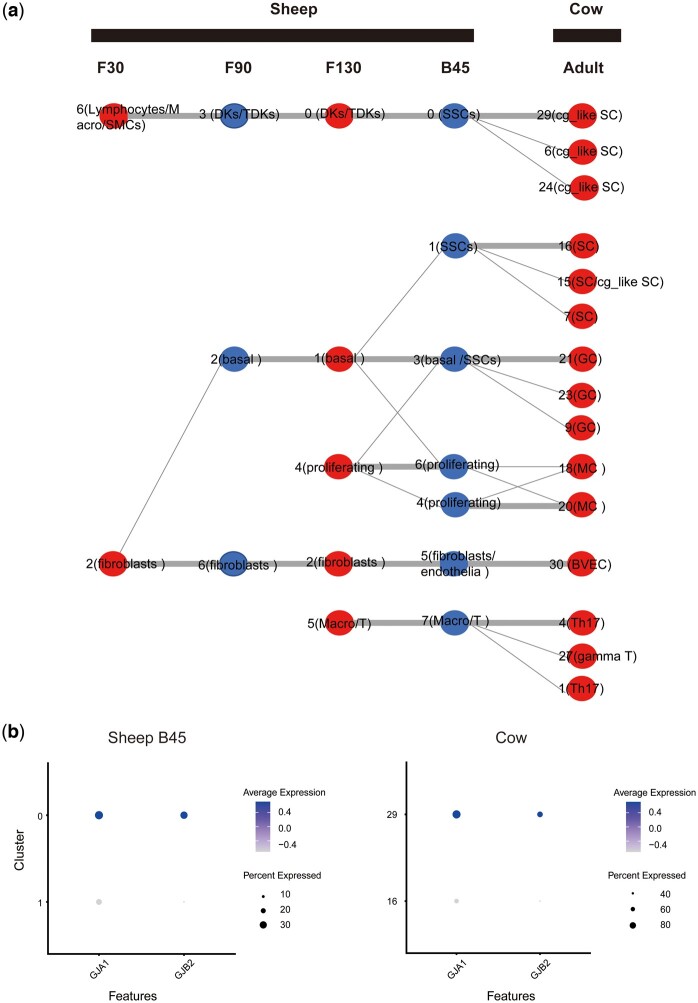
Alignment of sheep and cattle rumen single cell data at different developmental stages. (**a**) Diagram of conserved cell types and differentiation of the rumen in the cattle and sheep. ClusterMatch reveals six core conserved cell types and construct a differentiation map for rumen development. (**b**) Expression of gap junction protein genes with nutrient transport function in spinous cells of the 45-day-old sheep and the cattle. Cell types in sheep, basal: Basal cells; DKs: Differentiating keratinocytes; endothelial: epithelial cells; fibroblasts: Fibroblasts; keratinocytes: Keratinocytes; lymphocytes: Lymphocytes; macrophages: Macrophages; proliferating: Proliferating cells; SMCs: Smooth muscle cells; SSCs: Special spinous cells; T: T cells; TDKs: Terminally differentiating keratinocyte. Cattle cell types, BC: Basal cells; BVEC: Blood vascular epithelial cells; cDC: Conventional dendritic cells; cg_like SC: Channel-gap-like spinous cell; GC: Granule cell; MC: Mitotic cells; Th17: T helper 17 cells; SC: Spinous cells

Using scRNA-seq data from cattle and sheep, we obtained rumen conserved cell types and differentiation maps, as depicted in Fig. 5a. These clusters, obtained by ClusterMatch, were annotated and compared with manual annotation results (Supplementary Fig. S4a and b). We identified six conserved cell types (thick lines connect) shared between cattle and sheep datasets. Within the spinous cell type, manual annotation in the sheep data revealed only one type. However, leveraging the ClusterMatch model and annotation information from cattle, we discovered an additional conserved channel-gap-like spinous cell (cg-like SC) in the sheep data. In addition, based on the developmental data from sheep, we found that among the two types of spinous cells in cattle (cluster 16 and cluster 29), the gap-like spinous cells (cg_like SC, cluster 29) are more similar to F30’s cluster 6 (lymphocytes/macrophages/smooth muscle cells), while the spinous cells (cg_like SC, cluster 16) are more similar to F30’s cluster 2 (fibroblasts). Notably, compared to regular spinous cells, cg-like SC exhibited specific expression of GJA1 and GJB2 (Fig. 5b), encoding gap junction proteins responsible for nutrient transport (Goodenough and Paul 2009). This distinct cell type displayed a heightened capacity for the uptake of short-chain fatty acids (Wu *et al.* 2022), potentially contributing to efficient nutrient absorption in the rumen.

Regarding endothelial cells, blood vascular endothelial cells (BVEC) in cattle corresponded to mixed cell clusters of endothelial cells and fibroblasts in sheep B45 and fibroblasts at F30, F90, and F130. This suggests that rumen endothelial cells may originate from embryonic fibroblasts, which aligns with the findings of Li *et al.* (Li *et al.* 2013), and illustrates the potential of fibroblasts to transform into endothelial cells. ClusterMatch also matched cattle mitotic cells (MC) to sheep proliferating cells, both of which are proliferative. Finally, cattle T helper 17 cells (Th17) were matched by ClusterMatch to mixed clusters of sheep T cells and macrophages, indicating their presence as immune cells in the rumens of both species.

Overall, our results demonstrate the effectiveness of using ClusterMatch to analyze scRNA-seq data across species and different developmental time points, leading to the discovery of conserved cell types and differentiation relationships within the rumen.

## 4 Discussion

scRNA-seq technology allows biologists to gain the transcriptomic information from individual cells, uncovering cellular heterogeneity, gene expression patterns, and identifying cell type. Through the alignment of scRNA-seq data, including cross-species and cross-state alignment, etc., researchers can gain stable results by reducing the known and unknown sources of variability and invaluable insights into the understanding of developmental processes, evolutionary mechanisms, disease mechanisms, and the functional dynamics of biological systems (Arazi *et al.* 2018, Laughney *et al.* 2020, Shafer 2019).

Our ClusterMatch method deal with the known variability from high-dimensional and high-noise characteristics of scRNA-seq data, the batch effect between different datasets, and determining the resolution of clustering algorithms when defining and characterizing cell types. Our method outperforms the existing methods by overcoming the major difficulty in the alignment of scRNA-seq: (i) many algorithms have tunable parameters, and user-defined adjustments can produce very different results; and (ii) without knowledge of the cell type, it can be difficult to determine the quality of the clusters selected and whether the cells are under-clustered or over-clustered (Kiselev *et al.* 2017, Liu *et al.* 2021, Zappia and Oshlack 2018). Besides, existing approaches for alignment were mainly based on individual cell level alignment (Barkas *et al.* 2019, Haghverdi *et al.* 2018, Polański *et al.* 2020, Stuart *et al.* 2019), some approaches align scRNA-seq data at the cluster level (Korsunsky *et al.* 2019, Li *et al.* 2020, Welch *et al.* 2019). While cluster-level alignment in scRNA-seq data exhibits robustness against random fluctuations and may capture diverse cell states or types, as well as attractor states, the current methods for cluster-level alignment often rely on crude metrics such as cell numbers, lacking interpretability. Consequently, they provide limited information for subsequent cell type identification. ClusterMatch improves the cell cluster identification by both utilizing mutually consistent information and optimizing the multi-scale resolution parameter.

ClusterMatch model performed cluster-level alignment of scRNA-seq data based on the properties of attractor states that are less affected by random fluctuations and often correspond to distinct cell states (Bocci *et al.* 2022, Li *et al.* 2016). In this model, we used CCA to remove batch effects and project cells to a shared low-dimensional space. However, CCA, while effectively addressing batch effects, may inadequately consider local information, potentially obscuring biological specificity and leading to overcorrection issues (Luecken *et al.* 2022, Tran *et al.* 2020). To overcome this, we implemented multi-scale Louvain clustering for each dataset, utilizing DEG (differentially expressed genes) to represent clusters. On the one hand, DEG leverages local information, effectively avoiding overcorrection issues associated with CCA. On the other hand, by computing the correlation of clusters across different datasets at various scales, we provided appropriate clustering resolutions and the representation of DEG enhanced cluster interpretability. Furthermore, when determining the clustering resolution for a dataset, we used a stable matching algorithm that considered the clustering results of another dataset as a reference, which outperforms using the clustering results of a single dataset alone.

ClusterMatch can be extended to handle multiple datasets’ integration by transforming the multi-alignment problem into pairwise alignments. For instance, in scenarios such as comparing the rumen of sheep and cattle, we effectively integrate and align multiple datasets by performing pairwise comparisons based on their temporal order. However, without a natural developmental order, it may be necessary to perform pairwise integration and alignment for each dataset combination. Furthermore, since ClusterMatch operates at the cell type level rather than the individual cell level, it offers significant advantages in handling large-scale datasets by saving computation time and memory. While the number of cells in a dataset can be very large, the number of clusters (cell types) is typically much smaller, often only a few dozen. For example, the Xenopus and zebrafish embryo datasets (Song *et al.* 2023) comprise around 230 000 cells but only 36 cell types, and the natural killer cell dataset (Tang *et al.* 2023) contains approximately 160 000 cells but only 14 cell types. In addition, in Supplementary Fig. S5a, we present the running time of all the experiments in this study.

In our experiments involving human and mouse, human and macaque, and sheep and cattle, ClusterMatch required cross-species gene homology mapping. The specific process for converting homologous genes is detailed in the Section 2 under “Section 2.12.” Although some methods highlight the importance of incorporating one-to-one, one-to-many, and many-to-many orthologous genes for cross-species integration (Song *et al.* 2023, Tarashansky *et al.* 2021), the inclusion of one-to-many and many-to-many homologous genes was found beneficial only when they constituted a high proportion of the genes used in the integration, particularly for species with greater evolutionary divergence. ClusterMatch emphasizes multi-scale cluster-level alignment through stable matching of single-cell transcriptomic data. Because CCA requires one-to-one orthologous genes, we used only one-to-one homologous genes in our cross-species comparisons. To address the stability of ClusterMatch in handling different types of orthologs, we used human and macaque dLGN data. Given that CCA necessitates one-to-one orthologous genes, we averaged the expression levels of one-to-many or many-to-many orthologous genes to convert them into one-to-one orthologous gene expression levels. As shown in Supplementary Fig. S5b, our findings indicated that one-to-one orthologs accounted for 98.42% of the orthologous genes, one-to-many orthologs for 1.26%, and many-to-many orthologs for 0.32%. Despite these proportions, the annotation accuracy remained consistently high at 0.982 across all three scenarios. This demonstrates the robustness of ClusterMatch when the proportion of one-to-one orthologous genes is high.

Finally, our optimization framework has the potential to be extended to single-cell multi-omics data alignment. For instance, it can be applied to align scRNA-seq data with scATAC-seq data (Duren *et al.* 2018, Stuart *et al.* 2019), scRNA-seq data with scDNA-seq data, and scRNA-seq data with single-cell methylation sequencing data (Welch *et al.* 2019). In our future work, we plan to improve the cost function of ClusterMatch to incorporate data types from these emerging single-cell experiments. Furthermore, ClusterMatch is formulated as a nonconvex, nonlinear, and mixed integer optimization problem and this calls for more efficient algorithm.

## Supplementary Material

btae480_Supplementary_Data
