# Supplementary material for: ClusterMatch aligns single-cell RNA-sequencing data at the multi-scale cluster level via stable matching
Source: Bioinformatics. 2024 Jul 29;40(8):btae480. doi: 10.1093/bioinformatics/btae480 (PMC11520419; doi:10.1093/bioinformatics/btae480)
Supplement: btae480_Supplementary_Data [file btae480_supplementary_data.zip › ClusterMatch_Supplementary_Information.docx]

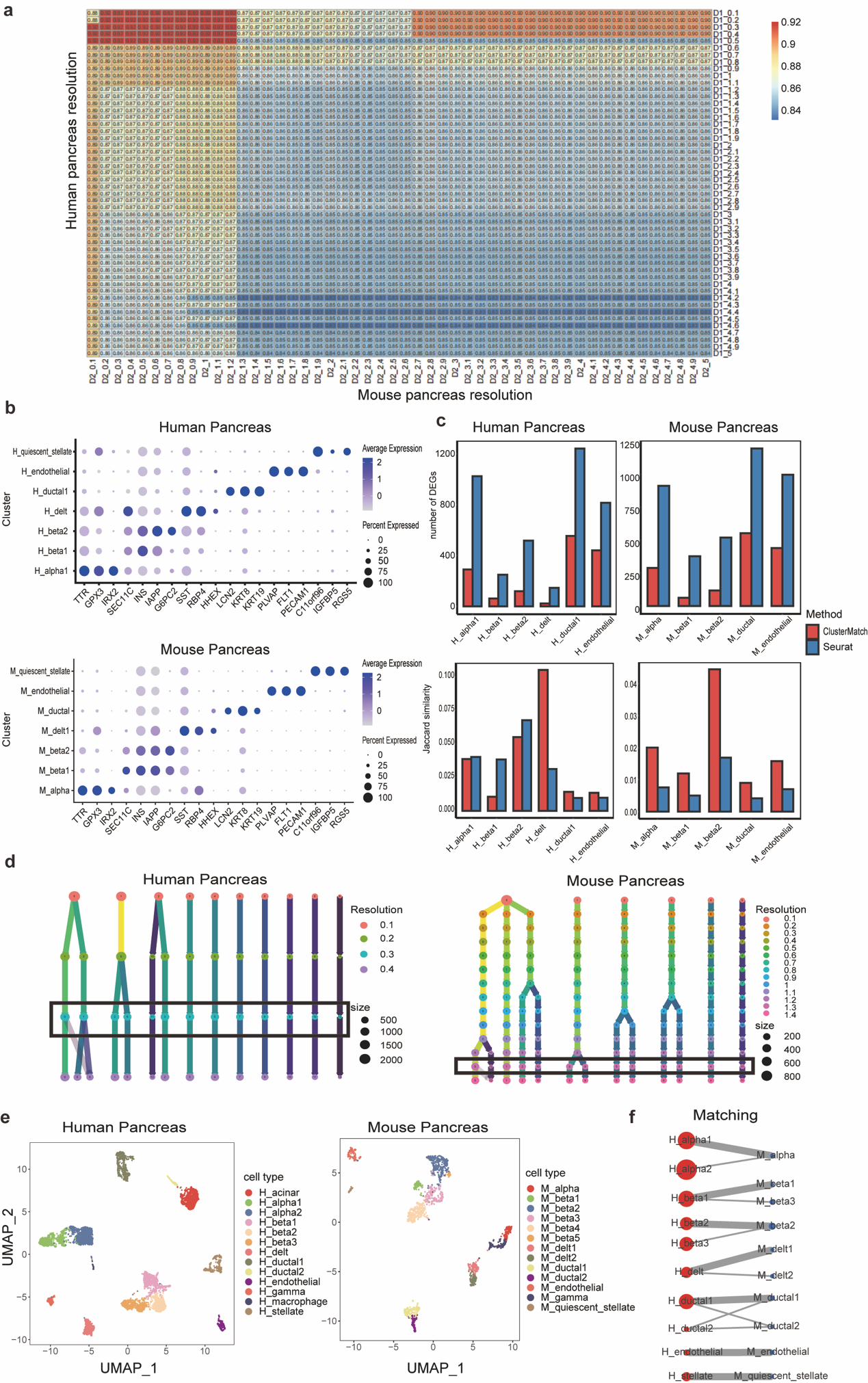


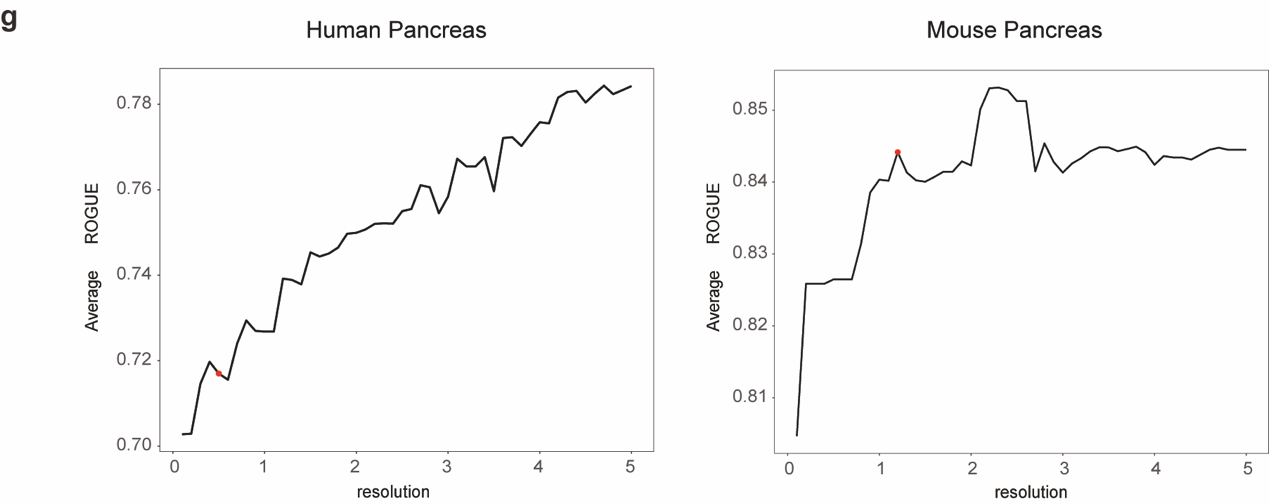


**Figure S1 a,** Average Pearson correlation coefficient of mutually most correlated clusters at different clustering resolutions from human and mouse pancreas data. **b,** Bubble heatmap showing the expression levels of the top 3 marker genes for the mutually most correlated clusters of the human (top) and mouse pancreas (bottom) data selected by ClusterMatch. **c,** Bar plot showing the number of differentially expressed genes and Jaccard similarity for ClusterMatch and Seurat V3. The Jaccard similarity is calculated using the marker genes from CellMarker2 database and the differentially expressed genes from ClusterMatch and Seurat V3. **d,** Clustree plot showing the size and the number of clusters for the human and mouse pancreas data at different clustering resolution parameter values. The black boxes correspond to the clustering results with the highest and stable resolutions of the Clustree method. **e,** UMAP visualization of human and mouse pancreas data, cells are colored by Clustree cluster membership (resolutions corresponding to black boxes in **d**). **f,** Alignment of human and mouse pancreas clusters by Clustree. **g,** The average ROGUE values for different resolutions using the Louvain method. The red dot represents the resolution corresponding to the ClusterMatch method along with its average ROGUE value.


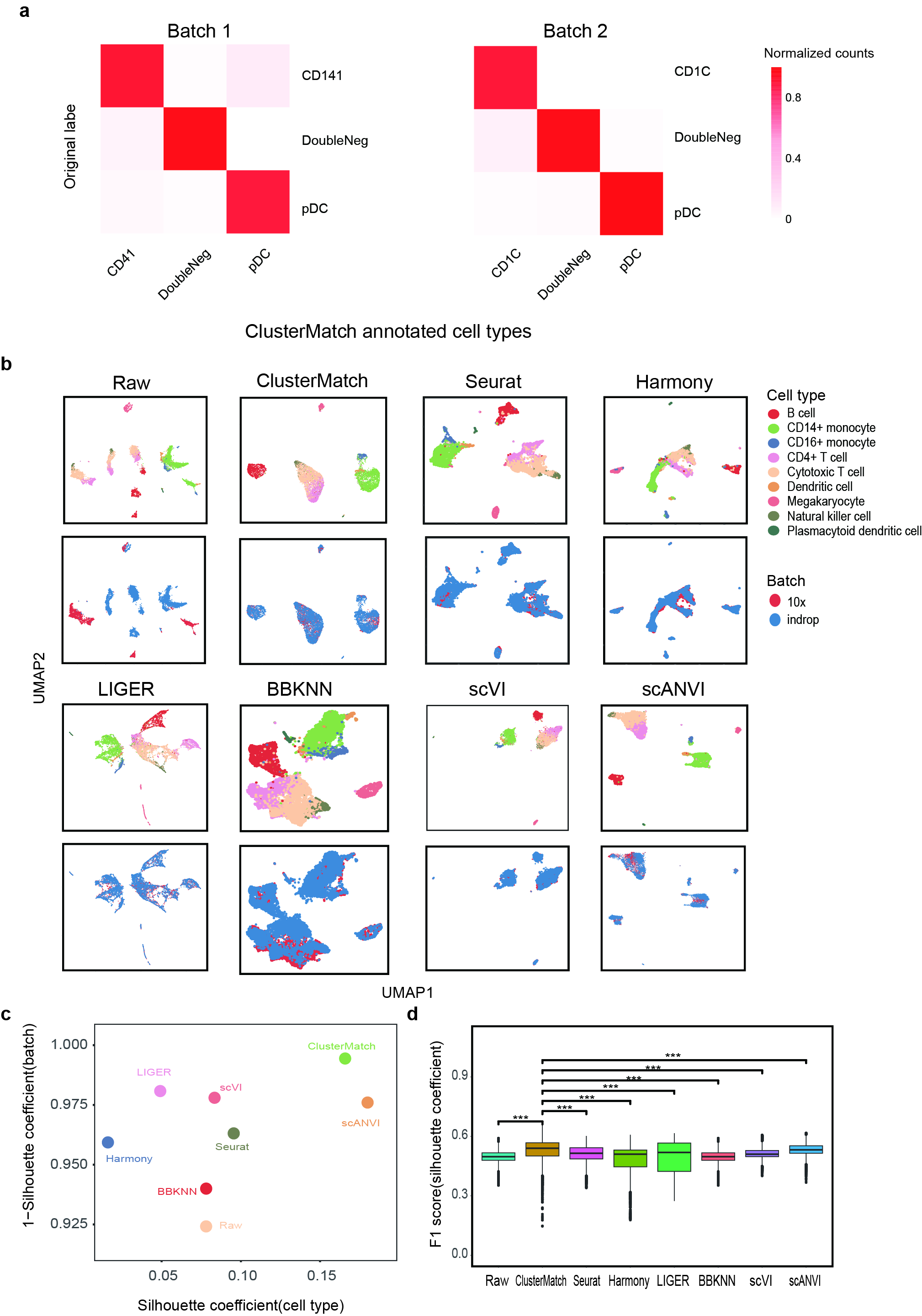


**Figure S2 a,** Agreement between the annotated cell types by ClusterMatch and the manually annotated cell types from Tran et al.. **b,** UMAP visualization of two integrated peripheral blood mononuclear cell (PBMC) datasets from Indrop and 10X technologies by ClusterMatch, Seurat V3, Harmony, LIGER, BBKNN, scVI and scANVI. The top panel is colored by the annotated cell types, and the bottom panel is colored by the technologies. **c,** Scatter plot of mean silhouette coefficients for ClusterMatch, Seurat V3, Harmony, LIGER, BBKNN, scVI and scANVI for PBMC data, where the x axis denotes the mean cell type silhouette coefficients, and the y axis denotes 1 minus mean batch silhouette coefficients; ideal outcomes would lie in the top right corner. ClusterMatch outperforms other four methods. **d,** Boxplots of F1 scores of silhouette coefficients for ClusterMatch, Seurat V3, Harmony, LIGER, BBKNN, scVI and scANVI for PBMC data. ClusterMatch significantly outperforms other four methods by a t-test analysis (p < 0.001), denoted by ***.


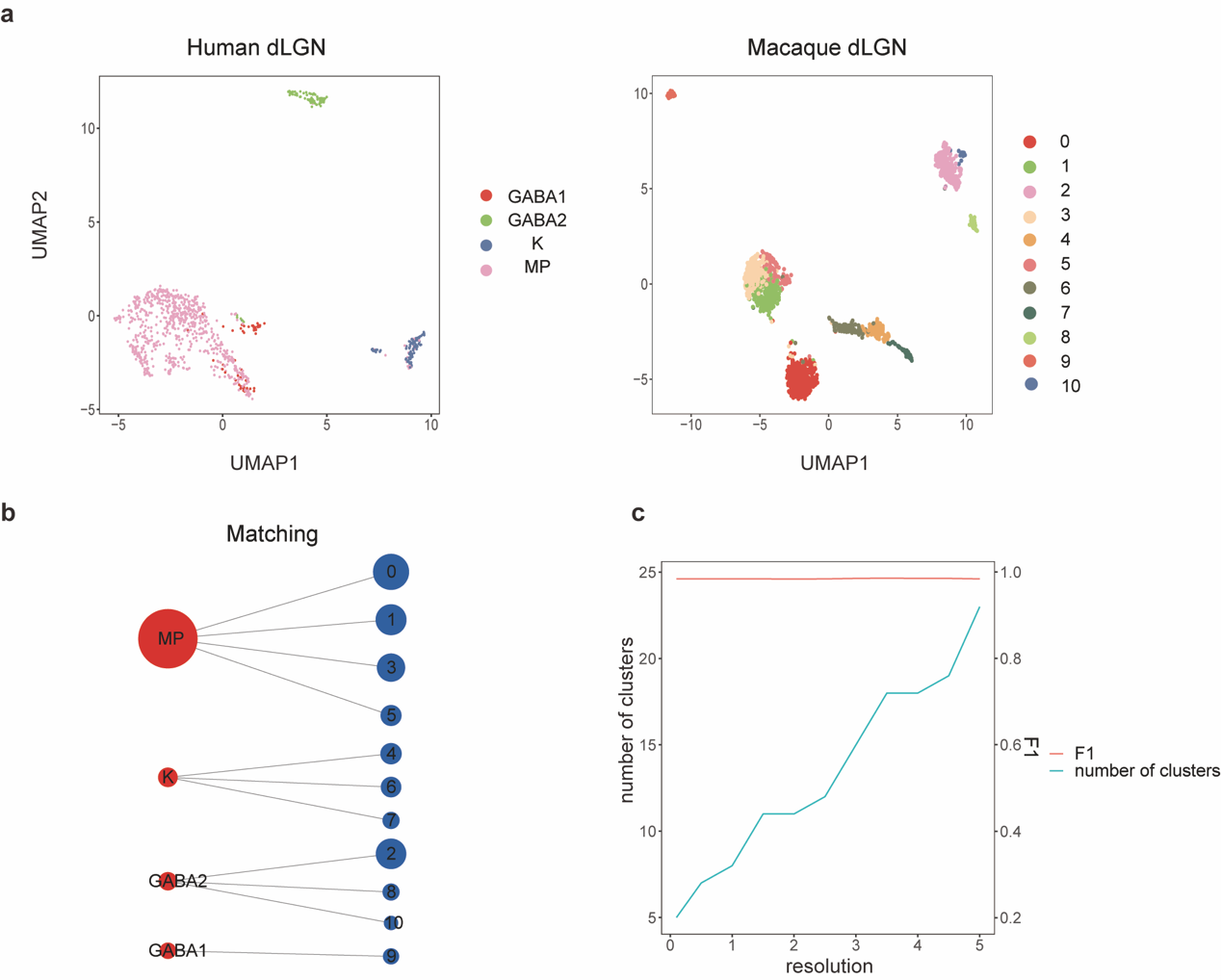


**Figure S3 a,** UMAP visualization of human and macaque dLGN data, cells are colored by manually annotated cell types (left) and ClusterMatch (right). **b,** ClusterMatch alignment of human and macaque dLGN clusters. **c,** The number of clusters (left y axis) and classification metric F1 scores (right y axis) for macaque data at different clustering resolutions, colored by F1 score and the number of clusters.


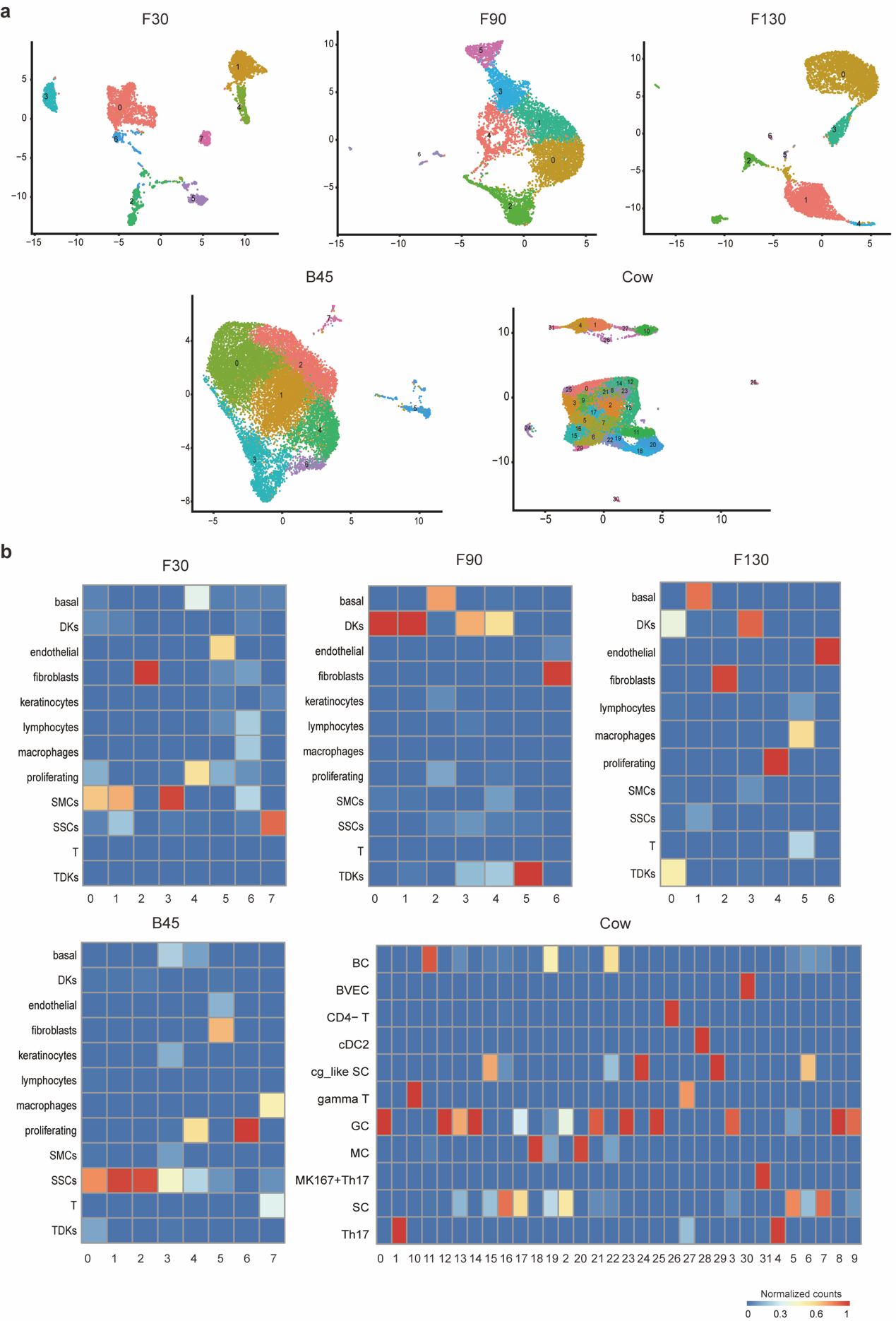


**Figure S4 a,** UMAP visualization of rumen data at F30, F90, F130, B45 developmental stages in sheep and in adult cattle, colored by cluster membership calculated from ClusterMatch. **b,** agreement between clusters by ClusterMatch and the manually annotated cell types. Cell types in sheep, basal: basal cells; DKs: differentiating keratinocytes; endothelial: epithelial cells; fibroblasts: fibroblasts; keratinocytes: keratinocytes; lymphocytes: lymphocytes; macrophages: macrophages; proliferating: proliferating cells; SMCs: smooth muscle cells; SSCs: special spinous cells; T: T cells; TDKs: terminally differentiating keratinocyte. Cattle cell types, BC: basal cells; BVEC: blood vascular epithelial cells; cDC: conventional dendritic cells; cg_like SC: channel-gap-like spinous cell; GC: granule cell; MC: mitotic cells; Th17: T helper 17 cells; SC: spinous cells.


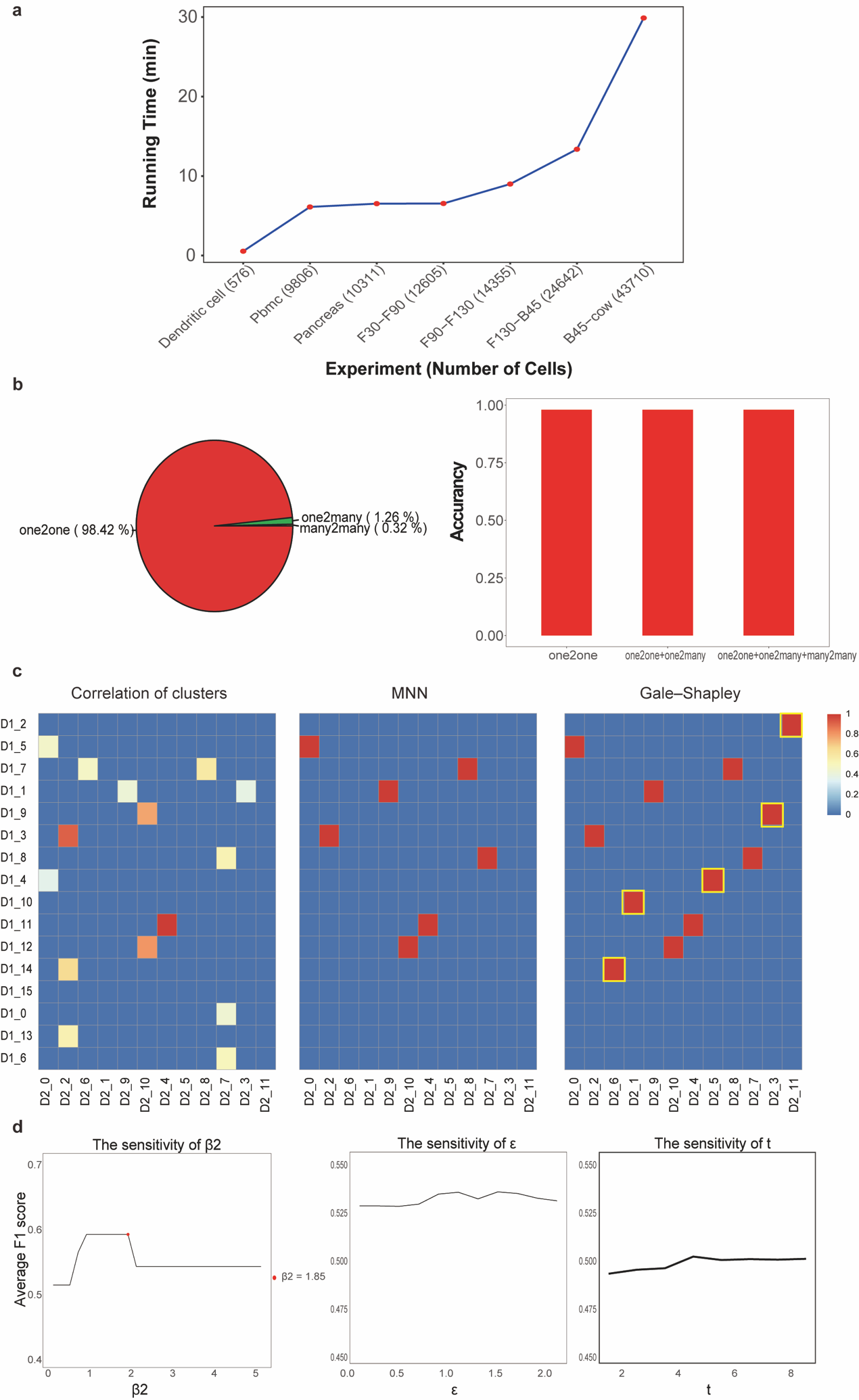


**Figure S5 a,** The runtime for each experiment using one core of an Intel Core i7-11700. **b,** Percentage of different types of orthologs (left) and annotation accuracy in three scenarios (right). **c,** Correlation between human and mouse pancreas clusters (left) and matching results using the MNN algorithm (middle) and the Gale–Shapley algorithm (right). **d,** ClusterMatch hyperparameter sensitivity analysis.
